# Rational Design and Delivery of Engineered Peptides: Toward a Unified Framework for Structure, Screening, and Application

**DOI:** 10.3390/biology15191754

**Published:** 2026-10-02

**Authors:** Qijun Xie, Haiying Ma, Biao Liu, Li Yu, Yang Fang, Lili Liu, Mingli Yan

**Affiliations:** 1 School of Biological Science and Medical Engineering, Hunan University of Technology, Zhuzhou 412007, China; m15575466097@163.com (H.M.); 18944914035@163.com (B.L.); yl230119860612@163.com (L.Y.); fangyang@hut.edu.cn (Y.F.); 2 Yuelushan Laboratory, Hunan Academy of Agricultural Sciences, Changsha 410125, China; ymljack@126.com; 3 Hunan Key Laboratory of Economic Crops Genetic Improvement and Integrated Utilization, School of Life and Health Sciences, Hunan University of Science and Technology, Xiangtan 411201, China

**Keywords:** engineered peptides, rational design, disulfide topology, phage display, peptide delivery, protein–protein interaction inhibitors

## Abstract

This review presents a three-tiered engineering framework that systematically addresses these barriers. First, we survey chemical and sequence-based stabilization strategies, including lipidation, backbone N-methylation, and cyclization, that convert labile natural peptides into protease-resistant scaffolds. Second, moving beyond generic stabilization, we dissect the rational design of effector-inhibitory peptides, with particular emphasis on disulfide bond topology engineering as a powerful means to lock binding-competent conformations, alongside structure-guided interface optimization. Third, recognizing that purely rational design often fails to correctly fold complex topologies, we discuss complementary structure-constrained screening platforms (phage display, yeast display, and in silico docking) that exploit sequence–conformation coupling to enrich functional structures from vast libraries. Critically, we argue that these molecular-level designs can only achieve in vivo efficacy when co-integrated with delivery systems, including lipidic, polymeric, and viral carriers, which together determine bioavailability, targeting, and controlled release. By weaving these layers into a unified framework, we highlight a paradigm shift from sequential optimization toward context-aware co-design, where peptide rigidity, flexibility, affinity, and carrier compatibility are balanced against specific therapeutic or industrial contexts.

## Simple Summary

Engineered peptides represent a promising class of functional molecules positioned between small chemicals and large protein biologics, offering unique advantages in therapeutic, agricultural, and materials applications. However, their practical use is severely constrained by inherent instability, rapid enzymatic degradation, and poor bioavailability. This review provides a systematic overview of current engineering strategies designed to overcome these limitations, organized around a three-tiered framework: chemical modifications that stabilize peptide backbones, structure-guided rational design that locks peptides into bioactive conformations, and high-throughput screening platforms that identify functional variants from vast sequence libraries. We further emphasize that molecular-level improvements alone are insufficient; effective in vivo application requires integration with delivery systems, including liposomal, polymeric, and viral carriers, that collectively govern bioavailability, targeting specificity, and controlled release. By weaving these interconnected layers into a unified perspective, this review highlights a paradigm shift from sequential optimization toward context-aware co-design, in which peptide rigidity, flexibility, affinity, and carrier compatibility are balanced against specific application contexts. The convergence of rational design, artificial intelligence-assisted prediction, and system-level formulation holds considerable promise for establishing engineered peptides as a versatile and transferable molecular toolkit, provided that challenges in scalability, predictability for non-canonical sequences, and regulatory adaptation are addressed in parallel.

## 1. Introduction

Peptide molecules, positioned at the interface between natural and artificial biological systems, are increasingly recognized as a central modality in next-generation functional molecule design and biopharmaceutical research. Owing to their high designability, excellent biocompatibility, and intrinsic molecular recognition specificity, peptides have demonstrated broad cross-disciplinary potential, spanning applications from antimicrobial defense and cancer therapy to food safety and organic electronic materials [1,2,3,4]. These attributes place peptides in a unique conceptual space between small molecules and proteins, combining synthetic accessibility with functional versatility.

However, despite the remarkable physiological activity and structural diversity exhibited by natural peptides, their practical application is often constrained by inherent physicochemical limitations. The predominance of linear backbones, low molecular weight, and high susceptibility to enzymatic degradation severely compromise peptide stability and bioavailability in complex physiological environments [5,6]. As a result, many native peptides fail to retain sufficient functional lifetime or efficacy under in vivo or industrial conditions, underscoring the need for systematic engineering strategies.

To address these challenges, the concept of engineered peptides has emerged, aiming to transcend the intrinsic limitations of natural peptide scaffolds through deliberate molecular design. By integrating chemical modification, sequence optimization, and structural reprogramming, engineered peptides are constructed to achieve enhanced stability, improved targeting capability, and tunable functional plasticity, thereby substantially expanding their application boundaries [7,8]. Importantly, recent advances suggest that peptide engineering is no longer a unidirectional effort toward maximal stabilization, but rather an evolving discipline that seeks to balance robustness with functional adaptability.

In this review, we begin by outlining the structural basis and biological functions of natural peptides, followed by a systematic discussion of recent advances in stability modification strategies, the design of effector inhibitory peptides, disulfide bond topological engineering, and peptide delivery systems. By integrating these developments, we aim to provide a unified perspective on how stability constraints, structural topology, and delivery context collectively shape peptide function, and to outline a forward-looking roadmap for the next generation of engineered peptide therapeutics and materials.

## 2. Applications of Peptides and the Challenge of Instability

Natural peptides have attracted widespread interest across diverse fields, from antimicrobial therapy and cancer treatment to food preservation and organic electronics, owing to their high biocompatibility, molecular recognition specificity, and synthetic accessibility [1,2]. For example, polylysine, a cationic peptide polymer, exhibits potent antimicrobial activity and biodegradability, making it a promising component of antibacterial hydrogels for wound healing [9,10]. In the food industry, polylysine has been shown to inhibit spoilage in tilapia, offering a “zero-additive” strategy for microbial control [11]. In materials science, the self-assembly and amphiphilic properties of natural peptides have enabled the fabrication of field-effect transistors, piezoelectric materials, and biosensors [12,13]. These examples underscore the remarkable functional diversity of natural peptides.

Peptide families currently dominating therapeutic development are particularly worthy of attention. Receptor agonists and antagonists, exemplified by GLP-1 receptor agonists, represent one of the largest and most commercially successful groups [14,15]. Antimicrobial peptides (AMPs) remain an active area of investigation despite historical challenges in formulation and delivery [16,17]. Cell-penetrating peptides (CPPs) have emerged as versatile vectors for intracellular delivery of otherwise impermeable cargo [18]. Disulfide-rich peptides (DRPs), including conotoxins, cyclotides, and knottins, leverage dense disulfide networks to form exceptionally rigid and proteolytically resistant scaffolds, with ziconotide serving as a clinically validated example [19,20]. Constrained peptides, including cyclic, stapled, and multicyclic structures, have gained particular traction as modulators of protein–protein interactions, a target class traditionally considered undruggable by small molecules [21,22].

The functional versatility outlined above is rooted in equally diverse structural architectures. Each peptide family exploits a distinct conformational logic: linear receptor agonists present sequence-defined epitopes for target engagement; antimicrobial peptides rely on amphipathic secondary structures, such as α-helices or β-sheets, for membrane disruption; disulfide-rich peptides achieve exceptional rigidity through dense crosslinking networks. Yet despite this structural diversity, all natural peptides share a common vulnerability: their backbone, composed of natural amino acids linked by standard peptide bonds, is inherently susceptible to proteolytic cleavage [23]. Moreover, many of the conformations that confer biological activity, particularly amphiphilic structures formed by alternating charged and hydrophobic residues, are marginally stable when isolated from their native context and can be readily disrupted by environmental changes [24].

It is precisely this combination of structural diversity and backbone vulnerability that creates a paradox: the same structural features that enable function in different peptide families also lead to instability. The small molecular size, linear backbone, and lack of stable tertiary conformation make most natural peptides highly susceptible to proteolytic degradation, resulting in short in vivo half-lives and low bioavailability [25,26]. This instability severely limits their pharmacological duration and practical application in complex physiological or industrial environments. Consequently, systematic engineering strategies are required to decouple, or at least balance, the inherent tension between functional structure and structural instability.

## 3. Engineering Strategies to Enhance Peptide Stability

To address the above challenge, researchers have developed a series of structural modification strategies aimed at prolonging the in vivo stability of peptides [27,28,29,30,31], enhancing their targeting capability, and reducing immunogenicity. Commonly employed strategies currently include: (1) chemical modifications (Section 3.1), which provide a versatile molecular toolbox, including lipidation, terminal capping, N-methylation, and cyclization, to directly reinforce the peptide scaffold against enzymatic attack; (2) sequence screening and in vitro evolution (Section 3.2); and (3) structure-guided rational design (Section 3.3) guided by structural information. These approaches function through distinct mechanisms to protect degradation-sensitive sites in peptides and optimize conformational stability [27,28,29,30,31], ultimately improving their biological efficacy.

### 3.1. Chemical Modifications: Enhancing Stability via Molecular Engineering

Chemical modification is one of the most direct and widely used strategies to stabilize peptides [32]. Given the distinct physical mechanisms involved, this section categorizes these approaches into two major classes: (i) steric shielding and end-group protection (exemplified by lipidation and terminal capping), which physically obstruct protease recognition through external hydrophobic barriers or end-group occlusion; and (ii) conformational entropy reduction (achieved through backbone N-methylation and side-chain crosslinking/cyclization), which rigidifies the peptide backbone into bioactive conformations that are inherently unfavorable for enzymatic cleavage. The representative chemical structures and a comparative summary of these four modification strategies are illustrated in Figure 1.

#### 3.1.1. Steric Shielding and End-Group Protection

A straightforward strategy to enhance proteolytic resistance involves erecting steric barriers that physically obstruct protease access to the peptide backbone without altering its intrinsic conformational preferences. This shielding effect can be achieved either through the covalent attachment of bulky hydrophobic moieties (lipidation) or through simple end-group capping.

Lipidation involves the covalent attachment of lipid molecules to the N-terminal amino group, lysine side chain amino groups, or cysteine thiol groups of peptides, thereby increasing their hydrophobicity and reducing their recognition by proteases [31]. The introduction of lipid groups not only disrupts the key hydrogen bond network required for proteolytic catalysis but also improves membrane affinity and solubility while reducing molecular aggregation. Such modifications can significantly prolong the half-life of peptides, reduce immunogenicity, and enhance bioavailability. For example, lipidated analogs of parathyroid hormone have been demonstrated to exhibit sustained activity both in vitro and in vivo [31]. Additionally, lipidation can promote targeted drug delivery, increasing its accumulation in specific tissues or cells. While lipidation provides a means of creating an outer structural “barrier,” the alteration in hydrophobic balance may also lead to reduced solubility or self-aggregation issues.

Fatty acids represent the most widely used lipid class for peptide modification, ranging from short-chain variants such as caprylic acid (C8) to long-chain variants like stearic acid (C18), with palmitic acid (C16) being one of the most prevalent modifications [33,34]. The site of lipidation critically influences peptide stability: N-terminal acylation blocks the peptide’s free N-terminus, reducing recognition by exopeptidases and thereby enhancing proteolytic stability. Side-chain lipidation, typically at lysine ε-amino groups, offers greater flexibility in modification site selection and can preserve the N-terminus for receptor binding while still conferring albumin-mediated half-life extension [33,35]. Notably, positional effects have been observed where shuffling fatty acid tails from the N-terminus to internal lysine residues can preserve activity against Gram-positive bacteria while increasing activity against Gram-negative strains and reducing cytotoxicity [35]. Furthermore, the chain length–stability relationship follows a bell-shaped distribution, with medium-chain fatty acids (C10–C12) often conferring the optimal balance of stability and selectivity, whereas excessive chain length (C16 and above) may promote peptide aggregation or hemolysis [33,35].

Complementing this bulk-scale hydrophobic shielding, terminal capping acts as a minimalist yet highly effective local barrier, which includes N-terminal acetylation and C-terminal amidation that protect peptides from aminopeptidase- and carboxypeptidase-mediated degradation, respectively. Quantitative degradation assays by Rozans et al. directly confirmed this protective effect: using peptide libraries with various N- and C-terminal functionalizations, the authors found that peptides containing free N-terminal amines were almost entirely degraded within 48 h, whereas simple terminal modifications significantly reduced or completely abolished nonspecific degradation, providing a quantitative benchmark for the end-group protection strategy [36]. These modifications are frequently combined with other stabilization strategies to achieve additive improvements in metabolic stability. For example, chemical optimization of vasoactive intestinal peptide receptor antagonists demonstrated that terminal modifications together with PEGylation and stapling can substantially improve in vivo persistence and antitumor efficacy in acute myeloid leukemia models [37].

#### 3.1.2. Conformational Entropy Reduction via Backbone and Side-Chain Constraints

In stark contrast to external steric shielding, a more sophisticated stabilization paradigm relies on reducing the conformational entropy of the peptide itself, thereby locking it into bioactive conformations that are sterically unfavorable for protease binding and cleavage. This entropic restriction can be imposed either on the backbone amide nitrogens (local torsional constraint) or via covalent side-chain crosslinks (global topological locking).

At the backbone level, N-methylation of backbone amide nitrogens restricts backbone flexibility, disrupts hydrogen-bonding patterns involved in protease recognition, and can markedly improve resistance to enzymatic degradation while enhancing membrane permeability [27,38]. The strategic incorporation of N-methylated residues has therefore become a widely used approach for improving the pharmacokinetic properties of therapeutic peptides. In addition to increasing proteolytic stability, backbone N-methylation can promote conformational preorganization and facilitate passive membrane permeation, as exemplified by cyclosporine and other bioactive cyclic peptides [38,39]. More recently, N-methylation has been applied to peptide inhibitors targeting the APP-Mint2 protein–protein interaction, where selective backbone methylation not only enhanced proteolytic stability but also improved binding affinity through reduced conformational entropy, achieving sub-micromolar inhibition of Aβ production in cellular models [40]. Similarly, N-methylation of the antimicrobial peptide apidaecin demonstrated that the effects of this modification are highly position-dependent: certain N-methylated analogs retained full antibacterial activity while gaining resistance to serum proteases, whereas other methylation sites led to complete loss of function, underscoring the importance of SAR-guided optimization rather than empirical N-methyl scanning [41,42]. These examples highlight that N-methylation must be deployed with positional selectivity guided by structure–activity data to avoid disrupting critical amide hydrogen-bonding networks essential for target recognition.

Moving from backbone-level to side-chain-level constraints, covalent cross-linking strategies provide an even more pronounced conformational locking effect. Disulfide bonds formed between cysteine residues represent one of the most common natural conformational constraints in peptides. By stabilizing β-hairpins, loops, and other folded structures, disulfide linkages reduce conformational flexibility and enhance resistance to proteolytic degradation [29,43]. Disulfide-rich peptides (DRPs), such as conotoxins and cyclotides, derive their remarkable stability and target specificity from densely interconnected disulfide networks. In a different application, cyclization of the antimicrobial peptide LR18 via a disulfide bond significantly increased its serum half-life and improved antibacterial activity against drug-resistant strains compared to the linear form, while also reducing hemolytic toxicity—demonstrating that disulfide constraints can simultaneously enhance both stability and safety profiles [44]. These studies collectively show that disulfide bonds not only confer proteolytic resistance but also actively shape the functional topology of the peptide, expanding both structural and functional repertoires beyond those of linear peptides. However, oxidative folding and correct disulfide pairing remain major challenges during synthesis and manufacturing, motivating the development of alternative covalent cross-links and disulfide surrogates [43,45].

Hydrocarbon stapling involves the installation of an all-hydrocarbon bridge between two olefin-containing amino acids through ring-closing metathesis. This constraint stabilizes α-helical conformations, reduces susceptibility to proteolysis, and can improve cellular uptake [46,47]. Stapled peptides have shown considerable promise against intracellular protein–protein interactions that are difficult to target with conventional small molecules. Recent advances in staple design have focused on improving aqueous solubility, biocompatibility, and pharmacokinetic performance while preserving helix stabilization [47]. For example, a double-stapled peptide named DSARTC was recently developed to simultaneously stabilize both α-helix and β-sheet conformations, enabling it to function as a peptide-based PROTAC that degrades the androgen receptor splice variant AR-V7. Compared to its linear counterpart, DSARTC exhibited enhanced proteolytic stability and improved cell penetration, and in an enzalutamide-resistant prostate cancer animal model, it effectively inhibited tumor growth and reduced AR and AR-V7 protein levels, demonstrating that staple engineering can be extended beyond canonical α-helical targets to more structurally complex and therapeutically relevant motifs [48]. More broadly, stapling has been applied to peptide inhibitors targeting the RAS-RAF interaction, where the hydrocarbon constraint enhanced antiproliferative activity in RAS-mutant cancer cell lines; however, the study noted that staple position and linker length required careful optimization to avoid disrupting key binding interactions, underscoring that stapling efficacy is highly context-dependent [49].

Beyond disulfide bonds and hydrocarbon staples, peptide stabilization can also be achieved through head-to-tail cyclization, lactam bridges formed between Lys and Asp/Glu side chains, and thioether cross-links such as lanthionine bridges. These approaches reduce conformational entropy, improve proteolytic stability, and often enhance receptor selectivity. The optimal cyclization strategy depends on the peptide sequence, desired conformation, and biological target [21,45]. However, it is important to recognize that these stabilization modifications are far from ‘one-size-fits-all’ solutions and are frequently accompanied by functional trade-offs. For example, while lipidation and N-methylation can significantly enhance protease resistance, the former may induce peptide aggregation or nonspecific adsorption due to the introduction of excessive hydrophobicity, whereas the latter may disrupt critical amide hydrogen-bonding networks, leading to substantial losses in binding affinity [32,36]. The current literature tends to emphasize successful stability improvements while providing insufficient quantitative analysis of concomitant bioactivity losses. This reporting bias makes it challenging to select optimal modification combinations for a given peptide in practice. Future studies should therefore establish more systematic structure–activity relationship (SAR) databases to delineate the tipping point between stabilization gains and functional compromise, rather than merely accumulating modification strategies.

### 3.2. Sequence Optimization and High-Throughput Screening: Leveraging Natural Selection Principles to Enhance Degradation Resistance

While preserving the activity of natural peptides, sequence screening and in vitro evolution can yield molecules with significantly enhanced resistance to proteolytic degradation. Typical methods include mRNA display and phage display technologies.

For instance, the “two-step in vitro selection strategy” proposed by Howell et al. [28] first uses proteases to degrade the initial library, screening for peptide sequences with anti-degradation capability. This is followed by a positive selection step via mRNA display, ultimately obtaining peptide molecules with 100–400 times improved anti-protease activity. This strategy substantially prolongs the in vivo residence time of peptides, providing a viable pathway for the application of therapeutic peptides. The direct link between screening methodology and stability enhancement is further exemplified by the SUPR (scanning unnatural protease-resistant) peptide system developed by Fiacco et al. In this approach, an mRNA display library incorporating an unnatural amino acid was subjected to proteolytic challenge prior to affinity selection; the resulting SUPR peptides exhibited approximately 500-fold improvement in serum stability (t_1/2_ = 160 h) and up to 3700-fold enhancement in resistance to specific proteases compared to the parent sequence, while maintaining target affinity—demonstrating that direct selection pressure for protease resistance can yield stability gains far beyond those achievable through rational design alone [50]. Similarly, phage display technology also enables efficient target-oriented screening. By displaying peptide libraries on the surface of phages, researchers can directly isolate peptides with high affinity, and even with stable binding to specific proteins (see Section 4.2) [51].

As described above, mRNA display enabled the selection of stable peptide variants by directly linking genotype and phenotype. Subsequent methodological advances have substantially extended this stability-oriented framework (Figure 2). One important direction is the incorporation of non-canonical and rare amino acids, including D-amino acids, through genetic code reprogramming strategies [50,52,53,54]. It should be noted, however, that natural ribosomes inherently recognize and process D-amino acids with very low efficiency, making their incorporation technically challenging. The flexizyme system, flexible tRNA acylation ribozymes, was developed to address this limitation by enabling the preparation of diverse nonproteinogenic acyl-tRNAs without requiring natural aminoacyl-tRNA synthetase recognition [52,54]. These acyl-tRNAs can then read vacant codons created in the flexible in vitro translation (FIT) system, yielding peptides containing D-amino acids and other exotic building blocks. The above systems, including flexizyme, FIT, and mRNA display, have been incorporated into the RaPID (random nonstandard peptides integrated discovery) platform. For example, Imanishi et al. constructed a library of 10^12^ macrocyclic d/L-hybrid peptides containing five kinds of D-amino acids and performed RaPID selection against human EGFR, demonstrating that d/L-hybrid peptides can simultaneously achieve high proteolytic stability and unique structural diversity that is inaccessible to natural L-amino acid peptides [55]. Nevertheless, as noted by Goto and Suga [54], the multiple or consecutive incorporation of highly exotic amino acids such as D- and β-amino acids remains challenging, and this continues to be an area of active methodological development. Nevertheless, by expanding the chemically encodable alphabet, mRNA display libraries can access sequences with intrinsically enhanced resistance to proteolysis and structural destabilization, thereby embedding stability into a larger selectable molecular space.

In parallel, selection conditions have evolved from idealized in vitro environments toward more physiologically relevant or semi-in vitro regimes [54,56], in which peptides are challenged by proteases, competitive binders, or serum-like components during the selection process (Figure 2). Although indirect, these environmental constraints effectively bias enrichment toward molecules that retain structural integrity and function under complex conditions, further refining stability as a selectable trait. These developments indicate that mRNA display has evolved into a context-aware selection platform, in which chemical composition and selection environment are co-designed to guide the emergence of peptides with robust stability profiles under increasingly realistic conditions.

### 3.3. Structure-Guided Rational Design: Precision Deployment of Chemical Tools

In contrast to the screening-based discovery described above, which relies on stochastic library diversification and environmental selection, structure-guided rational design offers a hypothesis-driven counterpart: rather than introducing new chemical entities, this strategy focuses on how to deploy the chemical tools described in Section 3.1, such as cyclization, N-methylation, and lipidation, at precisely defined positions to maximize stability while preserving bioactivity. The core logic rests on dissecting the peptide into functionally critical (binding interface) and structurally vulnerable (termini, flexible loops, protease cleavage hotspots) regions, using co-crystal structures, NMR, or computational predictions as a blueprint.

For example, in the development of the PD-1 agonist peptide MN1.4, Carter et al. [30] implemented a sophisticated combination strategy: First, computational alanine scanning precisely identified the functionally critical residues (e.g., Tyr12 and Arg14) indispensable for PD-1 binding, establishing restricted zones for optimization. Subsequently, avoiding the key binding interface, targeted modifications were made at the non-functional termini and internal cleavage sites: (1) Cyclization was achieved by introducing a terminal disulfide bond, effectively resisting exoprotease degradation. (2) Peptide bond N-methylation was introduced at the identified trypsin cleavage site (Lys13-Arg14), fundamentally eliminating recognition by endoproteases. These modifications, based on a profound understanding of structural vulnerabilities, extended the serum half-life of MN1.4 from a few hours for the parent peptide MN1.1 to over 48 h, while fully retaining all its biological activity as a PD-1 agonist.

These modifications, guided by a detailed understanding of structural vulnerabilities, extended the serum half-life of MN1.4 from a few hours for the parent peptide MN1.1 to over 48 h, while fully retaining its biological activity as a PD-1 agonist. Importantly, this stability gain did not come at the cost of target binding: surface plasmon resonance binding studies confirmed that MN1.4 retained PD-1 binding affinity comparable to that of the parent peptide, and enzymatic cleavage assays verified that N-methylation at the Lys13–Arg14 site effectively blocked trypsin-mediated degradation. These results confirm that the modifications were successfully confined to non-essential regions, achieving a clear separation between stability-enhancing and binding-competent zones [30]. In another example, structure-guided rational design was applied to an antimalarial peptide derived from the Plasmodium parasite invasion machinery; through targeted substitutions informed by the peptide–receptor co-crystal structure, the authors enhanced proteolytic stability while preserving parasite invasion inhibitory activity, though they noted that certain stabilizing mutations at the binding interface led to reduced affinity, highlighting the need for careful positional selection [57]. More broadly, structure-guided design of macrocyclic peptides has been systematized through automated computational platforms that integrate target identification, conformational sampling, and chemical modification prediction, enabling the rapid generation of macrocyclic candidates with predefined binding modes and enhanced stability profiles [58]. However, these computational approaches remain dependent on the availability of high-resolution co-crystal structures, and their predictions for peptides containing non-canonical residues or complex disulfide topologies require experimental validation [58].

This work demonstrates that, guided by a clear structural biology blueprint, the comprehensive application of chemical tools, such as cyclization and the introduction of non-natural modifications, to perform “surgical” interventions on specific unstable sites can achieve a substantial increase in stability without compromising function. Thus, targeted mutation design provides a theoretical and methodological foundation for functionally reshaping natural peptides while preserving bioactivity and achieving stabilization. Nevertheless, even with these advances, effector peptides still face challenges such as high structural flexibility and low binding specificity, prompting researchers to further explore higher-order structural constraints and dynamic regulation mechanisms.

## 4. Rational Design and Mechanistic Elucidation of Effector Inhibitory Peptides

While the stability-enhancing strategies discussed in Section 3 effectively prolong peptide half-life, they do not, by themselves, address a more fundamental pharmacological challenge: how to engineer a peptide that not only survives in vivo but also outcompetes a natural ligand for a disease-relevant binding interface. This is the central objective of effector inhibitory peptide design, a class of molecules programmed to recognize and competitively block the functional interface of a pathogenic effector (whether of pathogen or host origin) whose interaction with a binding partner is essential for disease initiation or progression.

Achieving this functional precision demands design principles that extend well beyond proteolytic protection. Unlike globular proteins, linear peptides lack intrinsic conformational preorganization, suffering from a high entropic penalty upon binding and offering a limited contact surface. Moreover, the canonical amino acid repertoire restricts the chemical diversity available for interface optimization. To overcome these obstacles, the rational design of effector inhibitory peptides has evolved along three complementary and progressively refined trajectories: (i) backbone and side-chain conformational constraints to reduce the entropic penalty (Section 4.1); (ii) disulfide bond topological engineering to covalently lock the peptide into its binding-competent conformation (Section 4.2); and (iii) structure-guided interface design coupled with computational affinity maturation to optimize anchor residues and intermolecular interactions at the peptide-target interface (Section 4.3). Notably, while some of the chemical tools discussed below, such as N-methylation, cyclization, and stapling, overlap with those introduced in Section 3.1.2, the rationale and objectives are fundamentally distinct. The strategies introduced below aim to pre-organize the peptide into a binding-competent conformation and sculpt an interface capable of high-affinity, specific competition with a natural ligand.

### 4.1. Conformational Constraint and Backbone Modification

A common conformational constraint method involves the incorporation of non-natural amino acids or D-amino acids, which enhance stereochemical stability by restricting backbone rotational freedom [38,59]. Another approach involves chemical modification of the peptide backbone, such as introducing N-methylation sites or peptidomimetic bond structures, to improve stability and spatial rigidity without altering the key functional groups. These modifications not only enhance the thermodynamic stability of the structure but also reduce the entropic penalty associated with multiple conformations in solution, thereby improving targeting binding efficiency.

For example, studies by Zang et al. [60] and Schoggins et al. [61] demonstrated that moderate introduction of cyclization and N-methylation modifications in antiviral peptides effectively enhances the binding specificity, significantly improving in vivo efficacy. A more profound stabilization is achieved by applying global conformational constraints through covalent linkages that pre-organize the peptide into its active shape. Such a strategy was adopted by Ali et al. [47], who incorporated hydrocarbon “staples” into key α-helical structures (stapled peptides) to stabilize their spatial conformation, markedly increasing binding affinity for target proteins and cellular penetration capability.

More recent primary studies illustrate both the promise and the limitations of this approach. In a stapled-peptide campaign targeting RAS–RAF interactions, co-crystal complex structures and RAS–RAF interaction hotspots were used to identify four linear peptides—Raf-0 to Raf-2 and CRD-0—derived from the α-helical regions of the RAS-binding domain (RBD) and the cysteine-rich domain (CRD) of CRAF [49]. Raf-1 was selected for further modification using a hydrocarbon stapling strategy and N-terminal capping with stearic acid because it showed the highest binding affinity in an SPR assay. The resulting peptides Sraf-2-1 and Sraf-7-1 bound KRASG12C with Kd values of 3.56 μM and 2.62 μM, respectively, and demonstrated anticancer activity in CCK8 assays. They also reduced AKT phosphorylation, induced cancer cell apoptosis in a concentration-dependent manner, and inhibited cancer cell migration, with improved α-helix stability and cell permeability [49]. These findings support the view that stapling can simultaneously improve conformational stability and cellular activity. However, the binding affinity remained in the low-micromolar range, and the N-terminal stearic acid moiety may enhance membrane partitioning and introduce non-specific effects rather than solely improving target engagement. Moreover, direct evidence that these peptides competitively disrupt the RAS–RAF interface in cells, as opposed to indirectly suppressing downstream signaling, was not fully established in the research, and in vivo pharmacokinetic and pharmacodynamic data were not reported.

A complementary example comes from the discovery of non-natural macrocyclic Mcl-1 inhibitors [62]. Screening of DNA-encoded libraries yielded hit compound **1**, a 1.5 μM Mcl-1 inhibitor. A subsequent crystal structure demonstrated that compound 1 bound to Mcl-1 in a β-turn conformation, bringing the two ends of the peptide close together. This proximity enabled macrocyclization, which improved binding potency approximately 10-fold. Further exploration of a key hydrophobic interaction with Mcl-1 and the moiety engaging Arg256 led to additional potency gains. Optimized compound **26** is a <3 nM Mcl-1 inhibitor, while inhibiting Bcl-2 only at 5 μM and Bcl-xL at >99 μM, and induces cleaved caspase-3 in MV4–11 cells with an IC50 of 3 μM after 6 h [62]. This study is notable because it uses protein–ligand crystal structures and binding kinetics to guide design and understand potency gains, and it demonstrates selectivity within the Bcl-2 family. Nevertheless, the approximately 1000-fold gap between biochemical binding potency and cellular caspase-3 induction highlights persistent challenges in cell permeability, intracellular stability, or target occupancy. No in vivo efficacy data were reported.

Small stapled peptides have also been applied to KRAS. Starting from a binding pharmacophore identified from known RAS-binding peptides, a chemically divergent library of small stapled peptides was generated, yielding compounds with weak binding activity [63]. Exploration of structure–activity relationships and optimization led to low-micromolar binders of KRAS that block nucleotide exchange. This work demonstrates that even short 5–6-mer stapled peptides can engage a shallow RAS surface, but the relatively weak affinity and the absence of cellular and in vivo validation limit conclusions about therapeutic potential. In a related but mechanistically distinct approach, an α3β chimeric helix mimic was iteratively designed to covalently target oncogenic G12C Ras [64]. Several electrophiles were explored to optimize preferential alkylation of the desired C12 residue. The designed lead peptide modulated nucleotide exchange, inhibited activation of the Ras-mediated signaling cascade, and was selectively toxic toward mutant G12C Ras cancer cells. This covalent strategy may overcome the loss of non-covalent contacts that often accompanies mimicry of only a portion of a large binding interface. However, covalent targeting carries inherent risks: the covalent bond may dominate non-covalent interactions, increase the likelihood of non-selective binding, and require proteome-wide cysteine profiling to establish selectivity.

Through these structural constraint and backbone modification strategies, effector inhibitory peptides achieve not only greater spatial stability but also enable highly specific competitive binding in physiological environments, laying a solid foundation for subsequent structure–function optimization.

### 4.2. Disulfide Bond Topological Constraints: A Core Strategy for Stabilizing Binding-Competent Conformations

A more powerful strategy to lock peptide conformation is the introduction of disulfide bond topological constraints. Disulfide bonds can spatially fix specific regions of the peptide chain through covalent cross-linking, thereby forming stable and reproducible three-dimensional conformations [29,43,65].

This topological constraint not only enhances structural stability but also shapes specific surface features, enabling highly selective binding to target proteins. For instance, Ta et al. [66] and Sampoli Benitez et al. [67] found that the topological arrangement of disulfide bonds in EGF-like domains directly determines the spatial conformation and functional specificity of microdomains. Studies on archaeal proteins further revealed that cells establish independent disulfide bond formation systems by regulating the topological orientation of membrane proteins, significantly enhancing protein stability and catalytic activity [68,69]. In the field of drug design, Im et al. [70] and Victorio et al. [71] successfully constructed highly efficient inhibitory peptides targeting the Aβ42 protein through disulfide bond reconstruction. These peptides form stable antiparallel β-sheet structures via topological optimization, effectively inhibiting amyloid protein aggregation and illustrating the potential of topological optimization in designing functional inhibitors.

Thus, disulfide bond topological engineering is not only a crucial approach for stabilizing the structures of effector inhibitory peptides but also provides essential molecular support for designing peptide molecules with dynamic tunability and high specificity.

### 4.3. Target Structure Identification and Affinity Optimization

While strategies such as covalent conformational locking provide a universal means to enhance peptide stability primarily by restricting flexibility (thereby reducing the entropic penalty upon binding), achieving stronger target affinity demands a more direct, structure-informed approach, one that explicitly optimizes the intermolecular interactions at the peptide–protein interface. The targeting capability and binding affinity of peptides primarily rely on this precise structural complementarity during their interaction with target proteins. By leveraging high-resolution protein complex structures, computational analysis and spatial simulation of key binding sites can guide the rational design of inhibitory peptides [72,73]. Notably, the complete workflow of rational design often includes experimental validation (Figure 3).

In the design process, peptides are often treated as “customizable binding modules.” By mimicking the binding interface of natural ligands, researchers identify key amino acid residues capable of forming stable hydrogen bonds, hydrophobic interactions, or π–π stacking with the target [74]. Subsequently, energy minimization and molecular dynamics simulations are employed to optimize the spatial arrangement of these residues, achieving higher binding affinity and reduced off-target effects.

For example, in their study on the BTLA/HVEM system, Kuncewicz et al. [75] systematically identified key residues in the BTLA protein (such as R42 and R114, which mediate salt bridge formation) responsible for binding to HVEM, based on the crystal structure of the BTLA/HVEM complex. Building on this, they designed and synthesized a series of short peptides mimicking the BTLA binding interface. These peptides were indeed able to effectively compete with and inhibit the natural binding between BTLA and HVEM at both cellular and molecular levels. Regrettably, the affinity of these peptides (KD ~1.5 µM) remained lower than that of the natural BTLA protein. This highlights the challenges in such rational design, which may stem from limitations in structural biology knowledge or deficiencies in computational models.

Recent studies have extended this structure-guided approach to more complex protein–protein interactions. JMPDP-027, a potent cyclic peptide targeting the PD-1/PD-L1 interaction, was optimized by rational design and showed a potent restoring ability toward T cells with an EC50 of 5.9 nM, comparable to that of the anti-PD-1 monoclonal antibody pembrolizumab [76]. JMPDP-027 also exhibited high resistance to enzymatic hydrolysis in human serum, no observable toxicity, and potent in vivo anticancer activity comparable to that of a mouse PD-L1 antibody in a CT26 colon carcinoma model. These results are encouraging because they demonstrate that a cyclic peptide can achieve antibody-comparable functional activity and in vivo efficacy. However, the EC50 value reflects T-cell restoration rather than direct binding affinity. Direct target occupancy and selectivity data are needed before concluding that the effect is solely due to competitive PD-1/PD-L1 blockade.

A second PD-1/PD-L1 example, PD-1-0520, was developed through structure-based design starting from key fragments of PD-L1 that interact with PD-1 [77]. Five mimetic peptides were designed and further optimized into 22 cyclic peptide candidates. Through molecular dynamics screening and in vitro and in vivo validation, PD-1-0520 effectively inhibited the PD-1/PD-L1 interaction, restored the immune activity of tumor-infiltrating T cells, and achieved a 68% tumor inhibition rate in B16-F10 tumor-bearing mice without systemic toxicity. It promoted CD8^+^ T cell infiltration into tumors, upregulated activation markers, and remodeled the tumor immune microenvironment [77]. This study illustrates the power of integrating structure-based design, MD screening, and immunological readouts. However, this research does not report direct binding affinity or IC50 values, and the antitumor effect is immune-mediated, making it difficult to disentangle direct PD-1/PD-L1 blockade from broader immunomodulatory effects. Direct binding and selectivity assays against related checkpoint proteins would strengthen the mechanistic conclusions.

Structure-guided design has also been applied to the YAP–TEAD interaction. A 15-mer peptide incorporating non-natural amino acids selected by structure-based design was derived from the Ω-loop sequence 85–99 of YAP and was able to potently disrupt the YAP–TEAD interaction in the nanomolar range by targeting only one of the two important sites of interaction [78]. This is notable because the YAP–TEAD interface is extremely large, making small-molecule disruption difficult. However, the linear nature of the peptide raises questions about proteolytic stability and cell permeability, and targeting only one of two interaction sites may limit maximal inhibitory potency. In vivo validation was not reported.

Computational hotspot analysis has also been used to discover peptidomimetics that bind the RAS effector domain [79]. Computational analysis identified hot spots of RAS relevant for effector interactions and screened a library of peptidomimetics. Nine compounds were synthesized and assayed as RAS inhibitors in cultured cells. Most induced a reduction in ERK and AKT activation by EGF, a marker of RAS activity. The most potent inhibitor disrupted Raf and PI3K interaction with oncogenic KRAS, corroborating its mechanism of action as a protein–protein interaction inhibitor and validating the computational methodology. Improvement of one compound decreased the survival of pancreatic cancer cell lines harboring oncogenic KRAS [79]. This study is valuable because it links computational design to cellular signaling and cancer cell viability. However, no direct binding affinities, structural validation, or in vivo data were reported, and selectivity against wild-type RAS or other GTPases remains unclear.

Finally, Li et al. [80] systematically summarized the logic behind selecting anchoring residues in the design of “stapled peptides”: different types of cross-linking strategies (such as Lys-Asp lactam bridges, Cys-Cys disulfide bonds, and olefin metathesis-based cyclization) directly influence the helicity, rigidity, and membrane permeability of the peptide. By pre-organizing the conformation, these strategies significantly reduce the entropic penalty upon binding. This hierarchical anchoring design, based on structural insights, enables researchers to “tune” peptide constructs, customizing them for high affinity and favorable drug-like properties according to the interface characteristics of different targets.

In summary, the rational design of effector inhibitory peptides achieves precise control from the sequence level to the spatial structure by integrating multi-dimensional strategies such as conformational constraints, backbone modifications, and affinity optimization. Meanwhile, its theoretical limitations must be acknowledged. First, the scarcity of high-resolution co-crystal structures, particularly for membrane proteins or conformationally flexible targets, means that the ‘precision deployment of chemical tools’ is often based on homology models or low-accuracy computational predictions, which severely constrains design success rates [58]. Moreover, current rational design overemphasizes ‘pre-organization’ and rigidification; while this reduces entropic penalties, it may sacrifice the conformational plasticity that is essential for high-affinity peptide-target binding. Moderate structural flexibility can be a prerequisite for achieving high-affinity interactions. Therefore, the design philosophy should evolve from pure ‘rigidity maximization’ toward ‘dynamic stabilization of the functionally competent conformation’, a goal that demands predictive accuracy far beyond current computational chemistry capabilities.

## 5. Screening and Validation Platforms Based on Disulfide Bond Topology

Since fully rational de novo design relies solely on structural knowledge, its predictive power remains severely constrained by our limited understanding of sequence–structure relationships. Moreover, practical synthesis poses additional hurdles: the oxidative folding process is inherently complex and difficult to control, and sequence mutations often lead to misfolding or the generation of unintended isomers [81,82]. Consequently, in vivo or semi-in vivo selection strategies have emerged as a practical compromise between purely in silico design and empirical discovery. Platforms which enable selection under structural constraints efficiently screen the vast sequence space by directly leveraging the intrinsic sequence-to-conformation relationship, thereby identifying peptides with the desired topological structures and biological functions. Cell-based display screening systems and computer-aided virtual screening systems are two representative types of these platforms. As summarized in Figure 4, these platforms differ in principle, workflow, advantages, limitations, and applications.

### 5.1. Phage Display Screening Platforms

Unlike the phage display technology used for sequence optimization mentioned earlier, the focus of phage display here is on structure-constrained screening. When researchers have identified a promising functional structural module (such as an effector inhibitory peptide or a stable natural peptide scaffold), the next challenge is to screen for molecules from a large number of variants that can correctly fold into predetermined stable structures (e.g., specific disulfide bond topologies). Within this framework, the structural differences between peptides are relatively smaller compared to the former (as shown in Figure 5).

Recently, researchers have modified phage display systems to enable the presentation of more complex topological structures. For example, Wang et al. [83] developed a phage platform capable of displaying covalent macrocyclic peptide libraries. Moreover, Xiang et al. [84] introduced a facile on-phage cyclization strategy using ortho-phthalaldehyde (OPA), enabling the construction of a 10^9^-member cyclic peptide library and the identification of binders against therapeutically relevant targets including PTP1B, NEK7, and hKeap1 with micromolar potency, confirming the efficacy of this platform for rapidly obtaining active ligands [84]. These examples illustrate the growing versatility of phage display in selecting functional peptides with defined topological constraints.

Beyond linear peptides, phage display can also be applied to structurally constrained scaffolds. Knottin is a highly stable microdomain rich in disulfide bonds, characterized by three disulfide bonds that form a stable “knot” structure. This structure allows Knottin to maintain its structural integrity under various environmental conditions. For instance, in the study by Souriau et al. [85], Min-23, a 23-residue peptide containing the cystine-stabilized β-sheet motif and representing one of the smallest autonomous folding units described to date, was used to construct a phage library of over 10^8^ clones by inserting random sequences into its β-turn region. Screening against seven different targets led to the isolation of 21 new specific binders, confirming the scaffold’s capacity to generate diverse binding specificities [85]. In another example, pH-controlled stepwise cyclization has been developed to enable phage display of heterobridged bicyclic peptide libraries, allowing the generation of bicyclic peptides with two distinct ring sizes and enhanced target binding properties [86]. More recently, a proximity-driven site-specific cyclization strategy was reported, which enables the efficient cyclization of phage-displayed peptides with high conversion rates, thereby increasing the effective library size and facilitating the discovery of cyclic peptides with improved stability and affinity [87].

However, the limitations of phage display are also evident: it is primarily suitable for displaying small proteins or short peptides and is less applicable to protein structures requiring complex folding or cofactor involvement [88,89]. Nevertheless, this platform remains an ideal tool for screening effector inhibitory peptides with stable disulfide bond topologies, given their relatively small size and simple folding requirements. A notable example of the therapeutic potential of phage display-derived constrained peptides is the identification of fibrin-specific peptides for imaging applications; through phage display screening, peptides with high specificity for fibrin were obtained and subsequently conjugated to imaging probes, demonstrating the translational relevance of phage display in diagnostic applications [90].

### 5.2. Yeast Display Screening Platforms

In parallel, yeast display provides a complementary screening platform for more structurally complex peptides, operating under conditions that more closely resemble the eukaryotic cellular environment while supporting post-translational processing and quantitative fluorescence-activated cell sorting (FACS). Yeast display technology utilizes eukaryotic host cells as an expression and presentation platform, fusing peptides or proteins to the cell surface while retaining complex post-translational modification and folding mechanisms [91,92]. Compared to phage systems, yeast display is more suitable for screening peptide molecules with multiple disulfide bond topological constraints, as it possesses a complete oxidative folding system and chaperone network within the cell.

Xu et al. [93] highlighted that the yeast display system offers significant advantages in protein processing, secretion, and folding fidelity, enabling the display of more complex polypeptide structures while maintaining their native conformation and activity. In parallel, recent platform-level innovations have expanded yeast display beyond conventional receptor–ligand screening (Figure 6). For instance, Huisman et al. [94] developed a novel yeast “peptide display” platform, which involves expressing MHC-II in insect cell lines first, followed by high-throughput display of peptide libraries in yeast, and then externally adding MHC-II. This strategy circumvents the folding bottleneck of MHC, enables universal screening across alleles, and transforms peptide-MHC binding into a tunable biochemical reaction with adjustable parameters. The utility of yeast display for discovering structurally constrained peptide therapeutics has been further validated by recent studies. For example, a yeast display platform was used to rapidly discover low-nanomolar macrocyclic peptide inhibitors of human angiotensin-converting enzyme 2 (ACE2); the selected macrocycles exhibited potent inhibitory activity (IC_50_ = 28 nM) and demonstrated that yeast display can effectively screen libraries of cyclic peptides with multiple disulfide constraints, yielding inhibitors with both high affinity and proteolytic stability [95]. Additionally, cystine-knot peptides based on the agouti-related protein scaffold have been engineered via yeast surface display for PET imaging applications; the optimized cystine-knot peptides exhibited high binding affinity for integrin targets and favorable pharmacokinetic profiles in vivo, illustrating that yeast display can be integrated with downstream formulation and imaging strategies for therapeutic development [96,97]. More broadly, modern yeast display platforms increasingly integrate quantitative sorting, conditional selection pressures, and downstream deep sequencing, allowing affinity, stability, and expression to be simultaneously optimized within a single evolutionary workflow [98,99,100] (Figure 6). When combined with FACS-based multiparameter gating, yeast display enables direct enrichment of peptide candidates that balance high binding affinity with correct folding and structural stability (Figure 6). As emphasized by Li et al. [92], this “structure–function-coupled” selection paradigm is particularly powerful for identifying peptide leads suitable for downstream therapeutic development, rather than merely high-affinity but unstable binders.

Given that both platforms are central to structure-constrained peptide screening, a brief comparison is warranted. Phage display offers substantially larger library sizes (10^9^–10^11^ vs. 10^8^–10^9^ for yeast), making it preferable for primary hit discovery, whereas yeast display enables quantitative FACS-based sorting and benefits from eukaryotic post-translational modification machinery, which is critical for screening disulfide-rich peptides [101,102,103]. Phage display, however, suffers from sequence biases due to the bacterial secretory pathway, while yeast display is limited by lower transformation efficiency and avidity effects arising from multivalent surface presentation [102,103]. These limitations can be mitigated through negative selection and deep sequencing monitoring for phage, and through multi-parameter FACS gating for yeast. Given their complementary strengths, hybrid strategies, employing phage display for initial hit discovery followed by yeast display for affinity maturation, have proven effective [102,104].

It is also noteworthy that both phage display and yeast display rely on genotype–phenotype linkage, meaning they inherently enrich sequences that are ‘easy to display’, i.e., well-expressed, properly folded, and stable, rather than those that are most biologically active. In phage systems, displayed peptides must tolerate the oxidative environment and protease attack of the bacterial periplasm, introducing intrinsic sequence biases. While yeast display overcomes some folding limitations, its screening environment still falls far short of recapitulating the complex mammalian physiological milieu. Consequently, many high-affinity peptides identified through in vitro screening fail in vivo due to an inability to cross cellular barriers, biofilms, or evade serum degradation. Therefore, screening strategies must evolve toward more ‘physiologically relevant’ competitive conditions, or incorporate counter-selection steps at later stages to eliminate false-positive clones whose stability depends on non-physiological conditions.

### 5.3. Computer-Aided Virtual Screening Platforms

With the development of artificial intelligence and computational biology, computer-aided virtual screening (CAVS) platforms are playing an increasingly prominent role in peptide drug design. These platforms can integrate sequence generation, conformational sampling, and molecular docking simulations to form an integrated computational pipeline from design to validation [105,106,107]. Recent studies have demonstrated the power of these approaches in practical applications. For instance, a machine-learning- and physics-based exhaustive hexapeptide screening model was developed to predict target-binding peptides; the model successfully identified peptide binders with high affinity, and subsequent experimental validation confirmed the predictions, demonstrating that computational screening can effectively reduce the need for exhaustive experimental library screening [108]. Similarly, cyclopeptide-based antitumor agents have been discovered through a pipeline combining design, virtual target screening, and experimental verification; the study showed that virtual screening could effectively narrow down candidate pools, though experimental validation remained essential for confirming activity [109].

Leveraging high-resolution structural information of target proteins, researchers can generate a large number of candidate peptides in virtual space and use molecular dynamics simulations to evaluate their disulfide bond topological stability and binding energy [110,111]. Research by Cui et al. [112] demonstrated that combining molecular docking with dynamic simulations can effectively predict stable binding modes between human proteins and inhibitory peptides, thereby significantly reducing the workload and cost of experimental screening.

Compared to traditional experimental screening, virtual screening offers advantages such as repeatability, speed, and cost-effectiveness, particularly in the early design stages where it significantly improves the efficiency of candidate molecule screening. Furthermore, this technology can be integrated with machine learning algorithms to provide multidimensional predictions of peptides’ physicochemical properties, conformational stability, and binding affinity, enabling dynamic optimization of design and validation. However, the reliability of this method has clear boundaries. First, its predictions heavily depend on the availability of high-resolution structures of the target proteins—a prerequisite often difficult to meet for many conformationally flexible targets or membrane proteins. Even when structures are available, early molecular dynamics force fields still exhibit deviations in accurately simulating the stability of key interactions such as disulfide bonds [113]. In response to these limitations, substantial methodological advances have been made over the past decade, including reparameterization of disulfide bond terms, improved treatment of torsional energetics, and the development of next-generation protein force fields [114,115,116]. Nevertheless, accurately capturing disulfide-rich peptide stability—particularly in highly constrained or solvent-sensitive environments—remains a nontrivial challenge for routine MD simulations.

Recent advances in artificial intelligence-based protein structure prediction, exemplified by AlphaFold [117] and related models (e.g., RoseTTAFold; [118])have substantially improved the ability to infer three-dimensional conformations from primary sequences. These approaches provide rapid structural hypotheses and facilitate structure-guided design even in the absence of experimental data. Building upon these predicted structures, molecular docking tools such as ClusPro and HADDOCK enable the computational modeling of peptide–target interactions. ClusPro is a widely used web server for protein–protein and protein–peptide docking that employs Fast Fourier Transform-based rigid-body docking and provides a specialized PeptiDock mode for global peptide docking [119,120]. HADDOCK, in contrast, is an integrative modeling platform that distinguishes itself by incorporating experimental data, such as NMR-derived restraints, cross-linking mass spectrometry, or mutagenesis information, directly into the docking calculations to guide complex prediction [121,122]. Together, these tools offer complementary strategies for evaluating binding modes and affinity, thereby enhancing the rational design of inhibitory peptides. Nevertheless, it should be noted that for engineered peptides, particularly those containing noncanonical residues, complex topologies, or context-dependent folding, prediction accuracy remains suboptimal, and dynamic conformational landscapes or functional states are often poorly represented by static predictions. Consequently, AI-based structural modeling complements but does not replace experimental selection.

In summary, through the synergistic application of phage display, yeast display, and computer-aided virtual screening, researchers can systematically screen peptides across different levels, from sequence to structure, and from structure to function. This provides a comprehensive technological pathway for the efficient development of engineered peptides.

## 6. Development of Delivery Systems and In Vivo Functional Realization

Even when engineered peptides with high stability and specificity are obtained through structural design and screening platforms, their therapeutic efficacy still depends on whether they can be efficiently delivered to the target site. Due to the susceptibility of peptide molecules to enzymatic degradation in body fluids, immune clearance, or physical adsorption, direct administration often leads to a rapid decline in their in vivo concentration. Therefore, constructing delivery vehicle systems that are highly compatible with the physicochemical properties of peptides is an essential prerequisite in realizing their in vivo function [123,124]. An ideal delivery system typically combines stability (primarily in the bloodstream), biocompatibility, low immunogenicity, targeted delivery capability, and controlled release properties (The peptide delivery modes of the three carriers are illustrated in Figure 7).

### 6.1. Liposomal Carriers: Integrating Biocompatibility with Intelligent Release

Liposomes are formed by the self-assembly of phospholipid bilayers, enabling the encapsulation of hydrophilic drugs within their internal aqueous core, while hydrophobic molecules can be incorporated into or associated with the membrane structure. Due to their structural similarity to cell membranes, liposomes exhibit excellent biocompatibility and tunability, making them one of the most widely used delivery systems for peptide-based drugs [125]. In recent years, liposomal design has shifted from passive encapsulation toward functionally programmed nanocarriers, incorporating stimuli responsiveness, surface engineering, and immunological modulation to better match complex therapeutic demands.

The advantages of liposomes are primarily manifested in two aspects: firstly, the lipid bilayer effectively shields the encapsulated peptides from the external enzymatic environment, thereby protecting them from degradation in the circulatory system; secondly, liposomes can be integrated with disulfide bond-stabilized peptides to form “stimuli-responsive systems,” where peptide release is triggered by redox conditions, enabling on-demand delivery [126]. Beyond redox responsiveness, contemporary liposomal systems increasingly exploit pH gradients, enzymatic activity, and membrane fusion dynamics to achieve spatiotemporally controlled peptide release, particularly within tumor microenvironments or inflamed tissues.

At the level of design philosophy, lipid composition is no longer treated as a passive container but as an active regulator of cargo behavior. By tuning lipid saturation, cholesterol content, and PEGylation density, modern liposomes can modulate membrane rigidity, circulation half-life, and cellular uptake pathways, thereby directly influencing peptide stability and bioavailability [127,128]. In addition, advances in lipid nanoparticle (LNP) engineering—originally driven by nucleic acid delivery—have begun to inform peptide delivery strategies, particularly for co-delivery systems and endosomal escape enhancement [129].

In practical applications, Park et al. [130] developed a CpG-DNA–peptide–liposome complex for the delivery of a TM4SF5 peptide vaccine targeting pancreatic cancer. This system induced robust TM4SF5-specific antibody responses in mice and significantly inhibited tumor growth in a BxPC-3 pancreatic cancer xenograft model, with the combination of peptide antigen, CpG adjuvant, and liposomal carrier proving essential for achieving therapeutic efficacy [130]. However, this efficacy was demonstrated only in a subcutaneous xenograft model, which does not recapitulate the desmoplastic stromal barrier of pancreatic cancer; moreover, CpG exhibits species-specific TLR9 activity, limiting direct translation of the observed immunogenicity to humans. Lee et al. [105] designed an active targeted delivery system using a fibronectin attachment protein-derived peptide as a targeting head conjugated to liposomes. This targeted formulation exhibited significantly higher binding and internalization in human bladder cancer cells (T24 and HT1376) compared to non-targeted liposomes, demonstrating the potential of peptide-directed liposomal delivery for improved tumor specificity [105]. Nevertheless, the bacterial origin of the targeting peptide raises questions about its specificity for human tumor cells, and the intracellular trafficking pathway—whether leading to lysosomal degradation or productive cytosolic delivery—remains unclear. Beyond these targeted approaches, liposomal formulations have been widely explored as peptide vaccine carriers. For instance, a nanoliposomal VEGF-R2 peptide vaccine elicited robust CD8^+^ T-cell responses and inhibited tumor growth in a murine melanoma model, though the magnitude of the immune response was highly dependent on adjuvant choice and peptide-loading density [131]. Similarly, HER2/neu-derived peptide nanoliposomes induced strong humoral and cellular immunity and conferred prophylactic efficacy against breast cancer; however, the study acknowledged that translating this prophylactic effect into a therapeutic setting would require overcoming the immunosuppressive tumor microenvironment [132]. More recent studies extend these concepts by integrating immune-adjuvant lipids or biomimetic membrane coatings, positioning liposomes not only as carriers but also as active participants in immune modulation and therapeutic synergy [133,134].

However, the encapsulation efficiency and stability of liposomal carriers are still influenced by the properties of the peptide. For highly polar or long-chain peptides, encapsulation efficiency may be suboptimal, and premature leakage can occur. These limitations have motivated the development of hybrid systems, such as polymer–lipid nanoparticles and lipid–peptide conjugates, which aim to combine the biocompatibility of liposomes with enhanced mechanical stability and programmable release behavior. While liposomal systems offer excellent biocompatibility and controlled release characteristics, their membrane integrity and structural stability are limited. To achieve higher delivery precision and programmable release behavior, researchers have further developed polymer-based carrier systems.

### 6.2. Polymer Carriers: Chemically Versatile and Environmentally Responsive

Polymer carriers have emerged as another important platform for engineered peptide delivery due to their flexible chemical structures and high tunability. Through molecular design, elements such as disulfide bonds, pH-responsive modules, or redox-responsive units can be incorporated to achieve controlled release in vivo [135,136,137,138].

Compared to liposomes, polymer carriers generally offer greater chemical and storage stability. They can load peptide molecules via physical encapsulation, electrostatic complexation, or covalent conjugation. In particular, polymer systems containing disulfide bonds remain stable under oxidative conditions during encapsulation but cleave and release their cargo in reductive environments (e.g., the cytoplasm or tumor microenvironment), enabling an “on-demand” targeted release mechanism. For instance, Han et al. [139] designed a self-assembling polymer, ATN-FFPFF-ATN, polymerized from phenylalanine dipeptide, ATN-161 peptide, and Pluronic^®^ F-127. The ATN-161 peptide segment targets integrin α5β1, which is highly expressed on certain breast cancer cells, enabling selective delivery to tumor sites. The carrier incorporates an acetal linkage between the peptide and Pluronic F-127, which is acid-labile and cleaves in the slightly acidic tumor microenvironment, leading to structural disassembly and pH-triggered release of the encapsulated drug. This system significantly enhanced drug accumulation and cytotoxic efficacy in breast cancer cells.

Further work by Koschek et al. [140] confirmed that the rigidity/flexibility of the polymer backbone critically influences its performance as a multivalent ligand scaffold. They found that rigid polymer scaffolds could form high-affinity complexes with proteins at low stoichiometric ratios, whereas flexible carriers like pHPMA and hPG were more prone to inducing protein cross-linking and aggregation under the conditions tested, indicating distinct biological application scenarios.

Moreover, polymer carriers can act synergistically with functional peptides: charged or hydrophobic residues within the peptide can engage in multiple interactions with the polymer backbone, stabilizing the composite system and promoting targeted aggregation [141]. This synergy, based on physicochemical compatibility, makes polymers a versatile and tunable carrier platform.

However, the primary challenges for polymer systems lie in their biocompatibility and the safety evaluation of their in vivo degradation products. To address this, researchers are exploring strategies that combine biodegradable polymers with natural matrices (e.g., chitosan, gelatin) to enhance safety while maintaining controllability. For example, redox-responsive hydrogels loaded with antibacterial peptides have been developed as controlled drug delivery systems for healing infectious wounds; the hydrogels exhibited sustained peptide release and antibacterial activity in vivo, though the study acknowledged that the long-term biocompatibility of the polymer degradation products requires further evaluation before clinical translation [137].

### 6.3. Viral Vectors: High-Efficiency Delivery and Intrinsic Penetration Properties

Unlike the two types of carriers mentioned above, viral vectors possess inherent cell-penetrating and nuclear delivery capabilities, enabling more efficient introduction of genes and peptide molecules. Their delivery efficiency is generally higher than that of liposomal and polymeric systems, making them particularly suitable for functional peptides that require precise delivery into cells or specific subcellular structures. However, in the context of engineered peptide delivery, viral vectors occupy a fundamentally different technological niche, as their native design logic is optimized for nucleic acid transport rather than the direct delivery of synthetic peptide molecules. As a result, the application of viral vectors in the delivery of engineered peptides still faces two major limitations. First, their inherent utility often mismatches the target scenario—viral vectors are typically suited for delivering nucleic acids or sequences for expression, rather than directly delivering synthetic peptides. When directly assembling or conjugating engineered peptides to the viral capsid, maintaining the correct conformation of the peptide during packaging, release, and infection poses a significant challenge. Peptides with stable structural elements, such as disulfide bonds, may demonstrate better compatibility in this regard [142,143]. Second, immunogenicity and regulatory constraints impose additional barriers, especially when repeated administration or systemic delivery is required. Even extensively engineered viral vectors, such as adeno-associated virus (AAV), may elicit neutralizing antibodies or trigger unintended immune activation, which limits their long-term applicability for peptide-based therapeutics.

The engineered application of viral vectors is primarily manifested in two aspects: On one hand, by genetically engineering the viral vector itself, its delivery properties can be precisely regulated. For example, Michelfelder et al. [3] modified the capsid proteins of adeno-associated virus (AAV) to display specific peptide functional elements on its surface, thereby enabling the viral vector to achieve targeted recognition and infection of specific cells, significantly enhancing the tissue specificity of gene delivery. Subsequent studies have extended this concept by systematically mapping permissive insertion sites and employing directed evolution to optimize capsid-peptide compatibility, improving both targeting specificity and transduction efficiency [144,145,146]. Bartel et al. established a directed evolution approach for AAV capsid engineering using random peptide display libraries, enabling the selection of variants with altered tropism [118]. Deverman et al. employed a Cre-recombination-based selection strategy to identify AAV variants capable of efficient blood–brain barrier crossing and widespread gene delivery to the adult mouse brain [119]. Ogden et al. further constructed a comprehensive fitness landscape of the AAV capsid, enabling machine learning-guided design of variants with enhanced production and transduction properties [120]. However, the selection conditions for capsid engineering are highly context-dependent and have not been systematically validated across serotypes. Notably, the marked drop in transduction efficiency observed when translating AAV variants from rodents to non-human primates underscores a major barrier to clinical application. On the other hand, viral vectors can be used to deliver genes encoding the target peptide, enabling endogenous expression within host cells. This approach circumvents the challenges of packaging and stability associated with direct peptide delivery. Studies by Abad-Fernandez et al. [147] utilizing cytomegalovirus (CMV) as a vector, successfully activated sustained immune responses through endogenous expression and delivery of peptide antigens, offering new strategies for vaccine development against complex viral diseases such as HIV.

More recently, the boundary between viral vectors and synthetic delivery systems has begun to blur. Hybrid strategies integrating viral vectors with polymer coatings, lipid envelopes, or chemically modified peptides have been explored to reduce immunogenicity, enhance targeting precision, and introduce stimulus-responsive control over peptide presentation or release [148]. For example, Pinto et al. demonstrated that polymer coating of adeno-associated viral particles effectively reduced neutralizing antibody recognition and enhanced transduction in the presence of pre-existing immunity, offering a promising strategy for repeat administration [4]. Although polymer coating reduces immunogenicity, it also alters viral surface properties and may compromise transduction efficiency in a cell-type-dependent manner. Furthermore, the long-term stability of coated vectors and the risk of polymer-specific immune responses upon repeat administration remain insufficiently characterized. These approaches reflect a broader trend in which viral vectors are no longer viewed as standalone delivery tools, but rather as modular components within multifunctional delivery architectures.

Despite the impressive delivery efficiencies demonstrated by liposomal, polymeric, and viral carriers in animal models, formidable challenges persist in translating these systems from laboratory scale to commercial production. Specifically, the batch-to-batch reproducibility of polymeric carriers and the long-term toxicity of their in vivo degradation products remain insufficiently characterized [109]. Although viral vectors offer superior efficiency, their high GMP production costs, potential oncogenicity, and acquired immune responses against the vector severely restrict their applicability in non-lethal or chronic diseases [120]. Moreover, existing delivery studies frequently overlook conformational changes that peptides may undergo upon carrier loading; the carrier microenvironment can induce unintended aggregation or loss of helicity, thereby markedly diminishing bioactivity. We must acknowledge that most currently designed ‘smart responsive carriers’ are overly idealized; their response specificity in complex physiological environments is far less robust than that observed in in vitro solution tests. Translating delivery systems from ‘proof-of-concept’ to clinical reality urgently requires addressing two fundamental bottlenecks: scalable manufacturing processes and long-term biosafety assessment.

Overall, viral vectors do not compete directly with liposomal or polymer-based systems in the routine delivery of engineered peptides. Instead, they define a specialized delivery paradigm that is uniquely suited for applications requiring intracellular gene-peptide coupling, long-term expression, or highly efficient cell-type-specific targeting [4,149]. Their future impact in engineered peptide therapeutics is therefore likely to emerge from hybrid and indirect delivery strategies rather than direct peptide encapsulation, positioning viral vectors as specialized-expanding rather than universal delivery platforms.

## 7. Prospects

### 7.1. Prospects

The application landscapes outlined above, spanning therapeutics, food safety, agriculture, and materials science, suggest a broader trajectory beyond domain-specific incremental advances. Collectively, they point towards the gradual emergence of peptides as a versatile and programmable molecular platform, in which sequence, conformation, and delivery context are co-optimized within an increasingly unified computational and experimental framework. In the coming decade, progress is unlikely to be limited to incremental improvements in stability or affinity; instead, it may witness the convergence of these fields into a shared engineering discipline, driven by several interrelated developments.

#### 7.1.1. Towards Context-Aware and Adaptive Peptide Functionalities

In the therapeutic domain, a key direction involves the transition from passive stabilization to intelligent, stimuli-responsive peptide systems. Rather than functioning solely as durable binders, next-generation peptides could be engineered to integrate sensing and actuation capabilities, discriminating between healthy and diseased microenvironments through combinatorial recognition of multiple biomarkers, such as pH gradients, redox potential, or specific protease activities [100,126]. This shift, from maintaining uniform stability to exerting activity preferentially at specific spatiotemporal coordinates, holds promise for redefining the therapeutic index, particularly for intracellular targets such as intrinsically disordered proteins and multi-protein complexes. In this context, cell-penetrating and endosomolytic designs that respond to endosomal maturation cues may render previously intractable targets more amenable to peptide-based intervention.

Beyond medicine, similar principles are beginning to extend into food safety and agricultural systems. Future antimicrobial peptides may no longer serve merely as static chemical substitutes, but instead could be designed as adaptive protective agents capable of sensing spoilage-associated volatile biomarkers or pathogen-derived quorum-sensing molecules, thereby activating their protective functions only upon detection of a threat [11]. Such strategies may enhance the protective capacity or efficacy of naturally occurring peptides; however, the current improvements remain primarily quantitative rather than transformative. Within existing technological frameworks, the design and manufacturing costs associated with implementing such intelligent response mechanisms remain substantial, and are likely to exceed the cost of simply increasing peptide dosage.

Consequently, these approaches may initially find commercial relevance only in specialized agricultural or food applications where stringent additive requirements justify higher costs. In conventional large-scale food production, their significance currently lies more in demonstrating future technological possibilities than in immediate industrial competitiveness. Similarly, in agricultural contexts with relatively permissive requirements, conventional small-molecule agrochemicals often retain structural advantages over peptides across multiple dimensions, including efficacy, persistence, and cost. Even with continued advances in recombinant production, cell-free synthesis, and formulation efficiency, engineered peptides will need to overcome substantial challenges before achieving broad competitiveness with established chemical alternatives.

However, these limitations do not imply an uncertain future for peptide technologies. The fundamental advantage of peptides lies not necessarily in outperforming small molecules in every application, but in providing a level of biological precision and regulatory flexibility that is difficult to achieve through conventional chemistry. The diversity of peptide-mediated biological functions is reflected extensively in nature; for example, subtle variations in peptide–protein interactions can produce finely tuned changes in receptor kinase phosphorylation patterns. Current limitations in mechanistic understanding and technological capability represent temporal barriers rather than permanent constraints on peptide potential.

Realizing this full potential, however, will likely require considerable time. In the near term, a more pragmatic development strategy may be to focus on applications where conventional small molecules remain ineffective or fundamentally limited. Viral diseases such as tobacco mosaic disease, for which effective chemical treatments remain largely unavailable, represent examples of niches where peptide-based therapeutics could provide uniquely valuable solutions.

In the field of materials science, peptides are expected to evolve from static structural templates into dynamic, stimuli-responsive components that transduce conformational changes into macroscopic property alterations, offering new opportunities for soft robotics, biosensors, and organic electronic interfaces [150,151]. This potential originates from the intrinsic nature of peptides as supramolecular building blocks: their self-assembly behavior and conformational adaptability naturally align with central themes in supramolecular chemistry. Particularly compelling is the fact that biological systems already provide extraordinarily sophisticated examples of how such supramolecular structures can be generated and regulated, from protein folding to the dynamic remodeling of cytoskeletal networks.

Nevertheless, just as supramolecular chemistry itself remains a frontier research field, the systematic exploitation of peptides as functional materials is still in its early stages. Fully realizing peptide-based materials will require an integrated research framework that simultaneously addresses sequence design, assembly regulation, environmental responsiveness, and macroscopic property characterization, rather than relying on isolated technological breakthroughs. Before such a framework becomes mature, a realistic transitional strategy may involve limited but strategic incorporation of peptide-responsive elements into conventional material platforms, enabling localized functional upgrades without requiring complete replacement of existing materials infrastructures.

#### 7.1.2. Data-Driven Design and Autonomous Closed-Loop Engineering

A second major development lies in the progressive integration of artificial intelligence with high-throughput experimentation. Currently, predictive capabilities for engineered peptides, particularly for disulfide-rich, macrocyclic, or non-canonical sequences, remain constrained by the predominance of natural protein data in training sets and by the systematic deviations of force fields in modelling constrained conformations [152]. Looking ahead, the emergence of peptide-specialized foundational models, trained on large-scale functional landscapes spanning antimicrobial activity, target engagement, carrier compatibility, and self-assembly propensity, may substantially improve predictive accuracy across diverse application contexts. However, the development of these models depends on a prerequisite that remains largely unmet: access to sufficiently large, diverse, and high-quality annotated datasets. These datasets must include not only successful sequences, but also failed designs, aggregation-prone variants, and other negative outcomes, precisely the types of information that remain scarce in the current field. The central limitation of predictive modeling may therefore gradually shift from insufficient model sophistication toward insufficient data diversity required to distinguish soluble from aggregating sequences, or stable from inactive designs.

These computational advances are likely to be complemented by autonomous closed-loop foundries, in which robotic synthesis, display-based selection (phage or yeast), deep sequencing, and machine learning iteratively refine design rules with minimal human intervention. Such platforms could compress multi-year optimization cycles into considerably shorter timeframes, while simultaneously generating standardized, open-access repositories of engineered peptide structures, stability profiles, and formulation outcomes [134,139]. More importantly, closed-loop systems naturally generate standardized structural and functional data, including both positive and negative outcomes, during operation, providing a potential mechanism for systematically addressing the aforementioned data gap. In this context, the accumulation of high-quality negative data may prove more urgent than further innovation in model architectures. The fundamental challenge in peptide prediction is increasingly becoming not simply that “models are insufficiently powerful,” but rather that models lack sufficiently comprehensive examples to learn the boundaries between favorable and unfavorable design spaces.

Once negative datasets are transformed from scattered experimental failures into systematically collected and openly available infrastructure, current predictive limitations may shift from fundamental barriers into manageable engineering challenges. From this perspective, one of the most important infrastructural priorities for the field over the coming years may not be the development of a single transformative algorithm, but rather the establishment of the first large-scale, openly accessible database linking engineered peptide structures with their functional outcomes.

#### 7.1.3. System-Level Integration: Co-Designing Peptide, Carrier, and Environment

Perhaps the most consequential shift will involve the gradual departure from sequential, siloed development workflows, in which stability engineering, affinity maturation, and carrier selection are conducted independently, towards integrated co-design platforms. It is increasingly recognized that peptide performance is not an intrinsic property but is strongly modulated by the surrounding delivery and deployment context [153]. Future engineering strategies are therefore expected to treat the peptide, the carrier system, and the target environment as a single functional unit from the outset.

Nevertheless, it is important to recognize that “co-design” currently represents more of a strategic vision than a mature engineering reality. Incorporating these three dimensions simultaneously into an optimization framework expands the search space from a largely one-dimensional problem into a highly multidimensional landscape, resulting in exponential increases in complexity and data requirements. Although advances in coarse-grained molecular dynamics and machine-learning-based formulation prediction are beginning to enable partial parallel optimization of sequence properties and carrier selection, substantial challenges remain before true integrated design becomes achievable.

For example, current simulation approaches still struggle to accurately describe, within practical computational limits, the coupled dynamics of peptide conformational transitions, carrier self-assembly behavior, and environmentally triggered release kinetics simultaneously. The interactions among these processes span multiple spatial and temporal scales, creating a fundamental challenge that cannot be easily addressed through incremental improvements in individual computational methods.

Despite these limitations, existing technologies are already sufficient to support a more limited but pragmatic form of co-design. Advances in coarse-grained molecular dynamics and machine-learning-based formulation prediction are beginning to enable rational carrier selection concurrently with sequence optimization, while redox-sensitive or pH-labile linkages offer means for spatiotemporally controlled release tailored to specific kinetic demands [126,137]. For therapeutic applications, this integration may yield peptide–carrier hybrids that not only protect and deliver but also actively contribute to target recognition and endosomal escape. In materials science, it may facilitate peptide-directed nanocomposites in which molecular assembly and macroscopic mechanical responsiveness are governed by the same design principles. For agricultural and food applications, such system-level thinking could lead to controlled-release granule formulations that protect peptides from UV degradation and enzymatic proteolysis while synchronizing release with crop growth cycles or spoilage timelines, provided that the cost–benefit balance can be favourably addressed through scalable manufacturing.

Within this framework, near-term objectives may differ substantially across application areas. In therapeutics, a priority may be the development of peptide–carrier hybrids that not only protect and deliver peptide cargo, but also actively participate in target recognition and endosomal escape, transforming carriers from passive delivery vehicles into functional components of therapeutic systems.

In materials science, a more immediately achievable goal may involve exploiting peptide self-assembly properties to direct hierarchical organization within nanocomposite materials, allowing molecular-scale ordering and macroscopic mechanical responses to be governed by a common design principle.

In agriculture and food applications, however, system-level integration will face the most stringent economic constraints. Controlled-release peptide formulations must simultaneously address ultraviolet degradation, enzymatic degradation, and synchronization of release kinetics with crop growth cycles or food spoilage timelines, while maintaining compatibility with large-scale manufacturing economics. Consequently, the primary optimization objective in these applications may not be maximizing performance, but rather achieving the minimum functional threshold within an acceptable cost increase. Importantly, this economic constraint should itself be incorporated as a design parameter rather than treated as a downstream consideration.

#### 7.1.4. Remaining Challenges and the Path Forward

Despite this optimistic outlook, several interdependent hurdles will need to be addressed to translate these concepts into practical realities. Importantly, economic barriers are not distributed uniformly across application domains, but instead exhibit substantial gradients depending on the intended use. The disparity is particularly pronounced in agriculture and food preservation, where peptide-based technologies continue to face significant cost disadvantages. Although peptide synthesis costs have declined substantially over recent decades, they remain considerably higher than those associated with conventional agrochemicals and bulk preservatives. Future progress in these sectors will therefore depend not only on improvements in biological performance, but also on advances in recombinant expression systems, enzymatic ligation strategies, and continuous-flow synthesis technologies capable of narrowing this economic gap.

By contrast, cost constraints are relatively less restrictive in therapeutic applications, particularly within high-value pharmaceutical markets. In these settings, economic feasibility is determined less by absolute manufacturing cost and more by whether peptides provide functions that cannot be achieved through alternative approaches. As discussed above, when peptides offer uniquely accessible biological capabilities, their value proposition is no longer governed primarily by direct cost comparison with conventional molecules.

A second major challenge concerns regulatory frameworks, which will need to evolve from molecule-centered evaluation toward system-level risk assessment approaches. Such frameworks must account for the dynamic behavior of responsive peptides, their degradation products, and their interactions with carrier materials. This transition should not be underestimated: it requires not only the development of new evaluation methodologies, but also the establishment of coherent regulatory pathways across pharmaceuticals, food additives, and environmental applications.

Third, the current fragmentation across scientific disciplines and application domains calls for strengthened collaborative infrastructures. Integrated training programmes, cross-disciplinary funding mechanisms, and community-driven data-sharing initiatives are likely to be necessary to bridge the cultural and methodological gaps between peptide chemists, formulation scientists, materials engineers, and regulatory experts.

In summary, while substantial scientific, technical, and economic challenges remain, the convergence of rational design, data-driven prediction, and system-level formulation holds considerable promise. If these interrelated efforts are sustained, engineered peptides may gradually establish themselves as an increasingly versatile molecular toolkit—capable of addressing distinct needs in human health, food safety, sustainable agriculture, and advanced materials, not as isolated solutions, but through a unified and transferable engineering philosophy. The realization of this potential, however, will require not only continued scientific ingenuity but also a collective commitment to engineering across traditional boundaries, with careful and continued attention to the practical constraints of cost, scalability, and regulatory acceptance.

## 8. Conclusions

In summary, the field of engineered peptides has evolved considerably, from isolated stability-focused modifications towards integrated strategies that concurrently address conformation, target selectivity, and delivery context. This review has highlighted that the central challenge lies not merely in extending half-life, but in achieving a context-dependent balance among rigidity, flexibility, affinity, and bioavailability. While the convergence of high-throughput screening, AI-assisted modeling, and precision formulation has created new opportunities, several bottlenecks, particularly the predictability gap for non-canonical sequences and the economic constraints facing agricultural deployment, will require continued attention. Progress in closed-loop evolution platforms, open-access structural repositories, and regulatory frameworks tailored to peptide complexity is likely to facilitate translational outcomes. If these efforts are sustained, engineered peptides may establish themselves as a versatile molecular toolkit that complements both small molecules and larger biologics across therapeutic, agricultural, and materials applications, provided that scalability and cost-effectiveness are addressed in parallel.

## Figures and Tables

**Figure 1:**
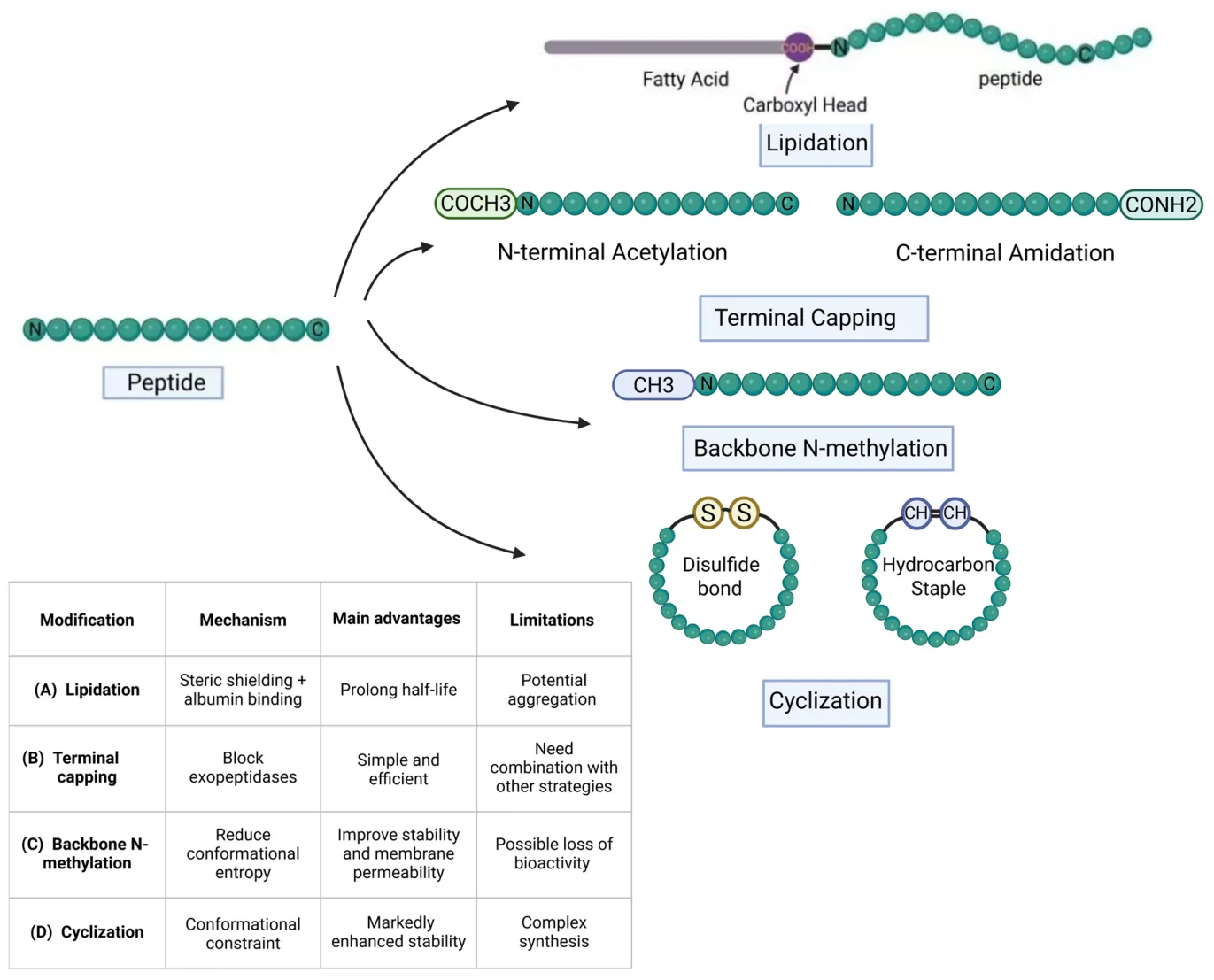
Representative chemical modifications for peptide stabilization. Schematic overview of four major strategies. (A) Lipidation attaches a fatty acid chain to the peptide, providing steric shielding and albumin binding; the main limitation is potential aggregation. (B) Terminal capping blocks exopeptidases via N-terminal acetylation and C-terminal amidation; often requires combination with other strategies. (C) Backbone N-methylation replaces amide hydrogen with a methyl group (N–CH_3_), reducing conformational entropy and improving membrane permeability; may compromise bioactivity. (D) Cyclization includes disulfide bonds (–S–S–) and hydrocarbon staples –CH=CH–, both imposing conformational constraint to enhance stability at the cost of synthetic complexity.

**Figure 2:**
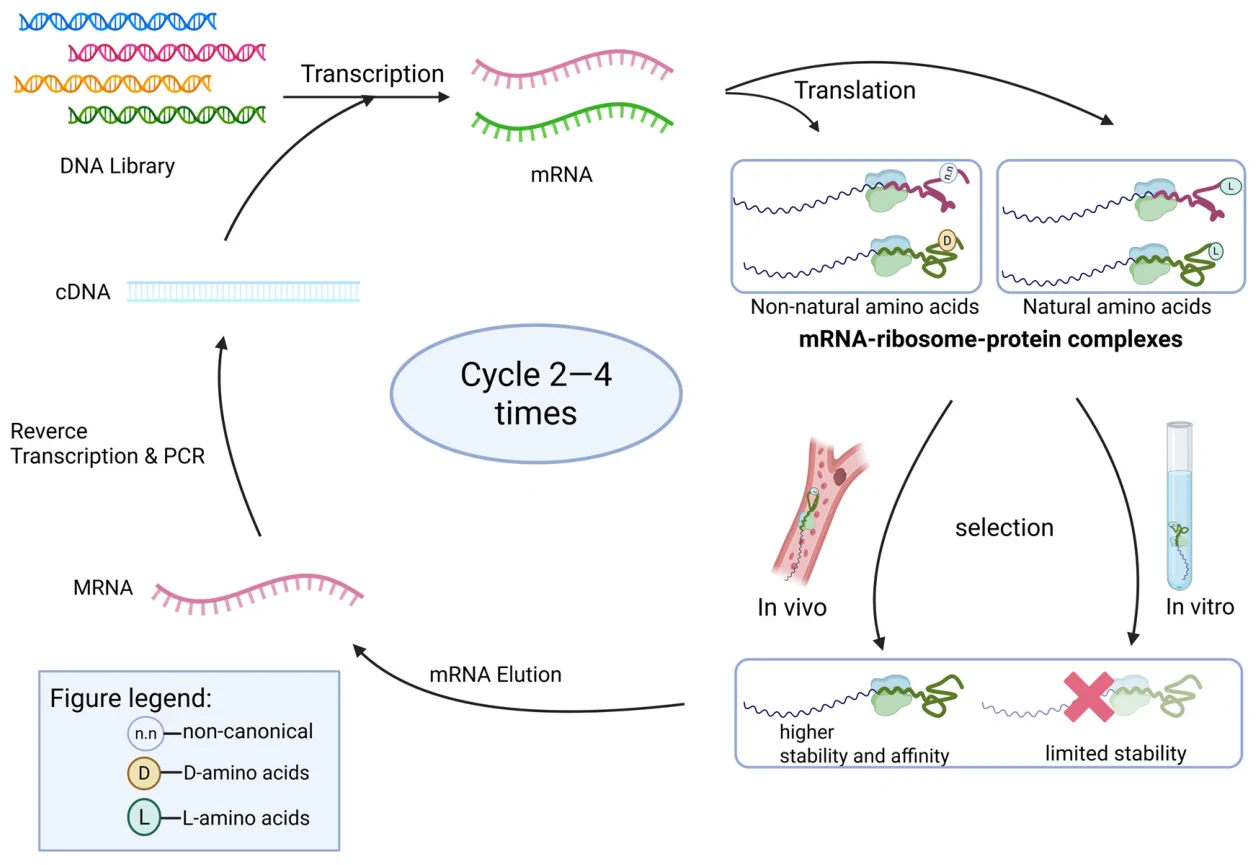
Context-aware mRNA display platforms for stability-oriented peptide evolution. Schematic overview of the iterative mRNA display cycle engineered for the selection of proteolytically stable peptides. The workflow initiates with a DNA Library, which is transcribed into mRNA and subsequently translated to form mRNA–ribosome–protein complexes, establishing a direct genotype–phenotype linkage. The translation process allows the incorporation of non-natural amino acids as well as standard natural amino acids (L-amino acids; as noted in the top right corner). The displayed peptide complexes are then subjected to a selection step under either in vivo (physiological) or in vitro (e.g., defined protease challenge) conditions (corresponding to the selection step). Following selection, the genetic information of the enriched complexes is recovered via mRNA Elution, and amplified by Reverse Transcription & PCR to generate a cDNA library. This entire procedure constitutes one selection round and is typically repeated 2–4 times (Cycle 2–4 times) to achieve optimal peptide enrichment.

**Figure 3:**
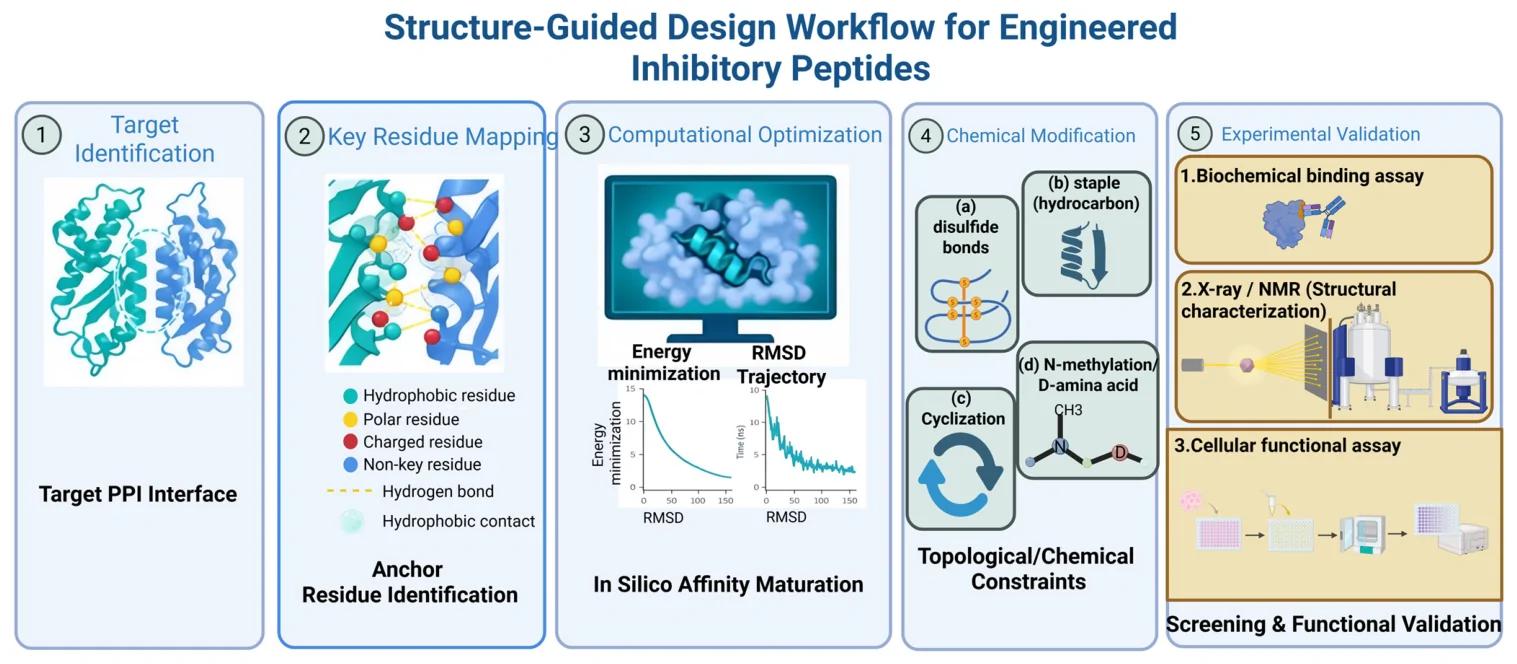
Structure-guided design workflow for engineered inhibitory peptides. The workflow comprises five stages: (**1**) target identification and PPI interface analysis; (**2**) key residue mapping and anchor residue identification; (**3**) computational optimization via energy minimization and molecular dynamics; (**4**) chemical modification through disulfide bonds, stapling, cyclization, N-methylation, or D-amino acid substitution; and (**5**) experimental validation by phage display, yeast display FACS, biochemical assays, and cellular activity assays.

**Figure 4:**
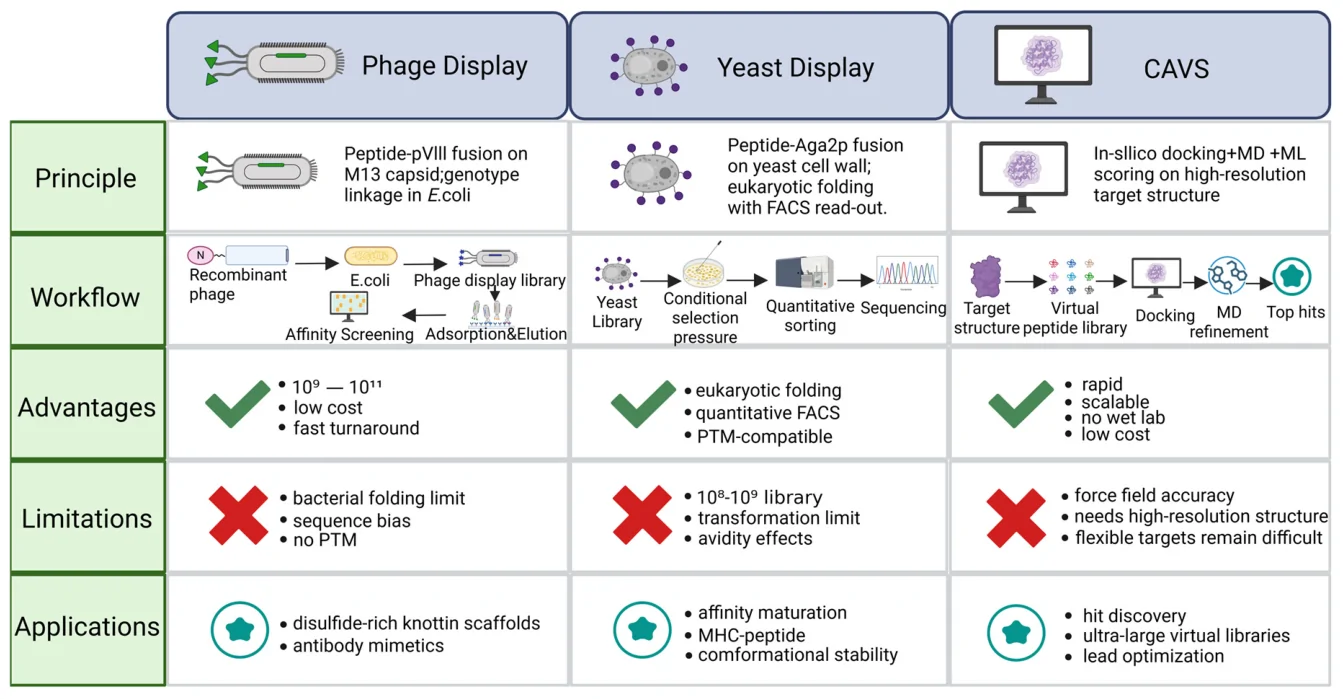
Comparative summary of screening platforms for structurally constrained peptides. Phage display, yeast display, and computer-aided virtual screening (CAVS) are compared in terms of principle, workflow, advantages, limitations, and applications. Phage display offers large library sizes and rapid hit discovery but is limited by bacterial folding and sequence bias. Yeast display supports eukaryotic folding and quantitative FACS but has smaller library sizes and avidity effects. CAVS enables rapid, low-cost virtual screening but depends on high-resolution target structures and accurate force fields.

**Figure 5:**
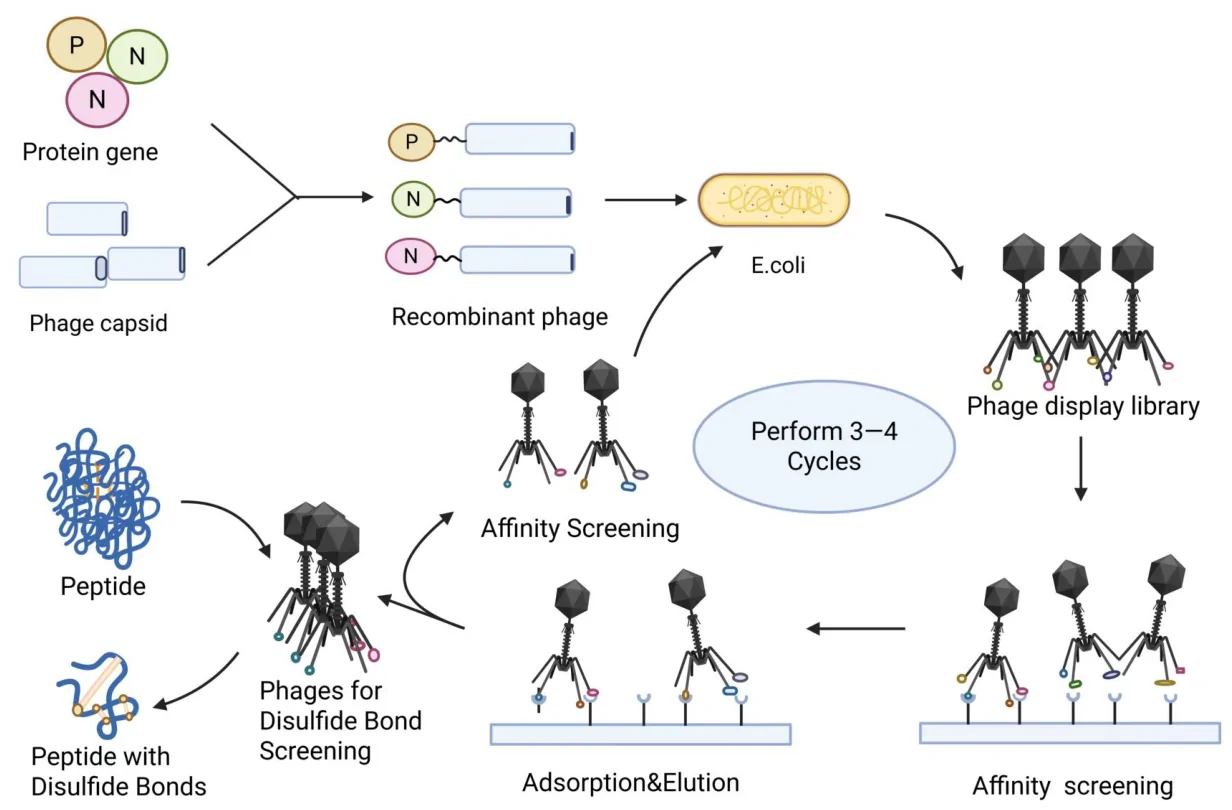
Structure-constrained phage display screening platform. Schematic representation of a phage display screening workflow for identifying structurally constrained peptides. The library construction initiates with the insertion of protein genes into the phage capsid, generating a recombinant phage (Different-colored dots represent displayed peptides). Following the amplification of these recombinant phages in *E. coli*, a diverse phage display library is established. This library is subsequently subjected to iterative rounds of affinity screening against immobilized targets, encompassing adsorption & elution to isolate binding variants. To enrich for high-affinity binders, the screening cycle is typically repeated for 3–4 cycles (noted as Perform 3–4 Cycles). Through this process, the platform effectively isolates specific Phages for disulfide bond screening or regular peptides, ultimately facilitating the acquisition of structurally stable peptides with disulfide bonds (as indicated in the output).

**Figure 6:**
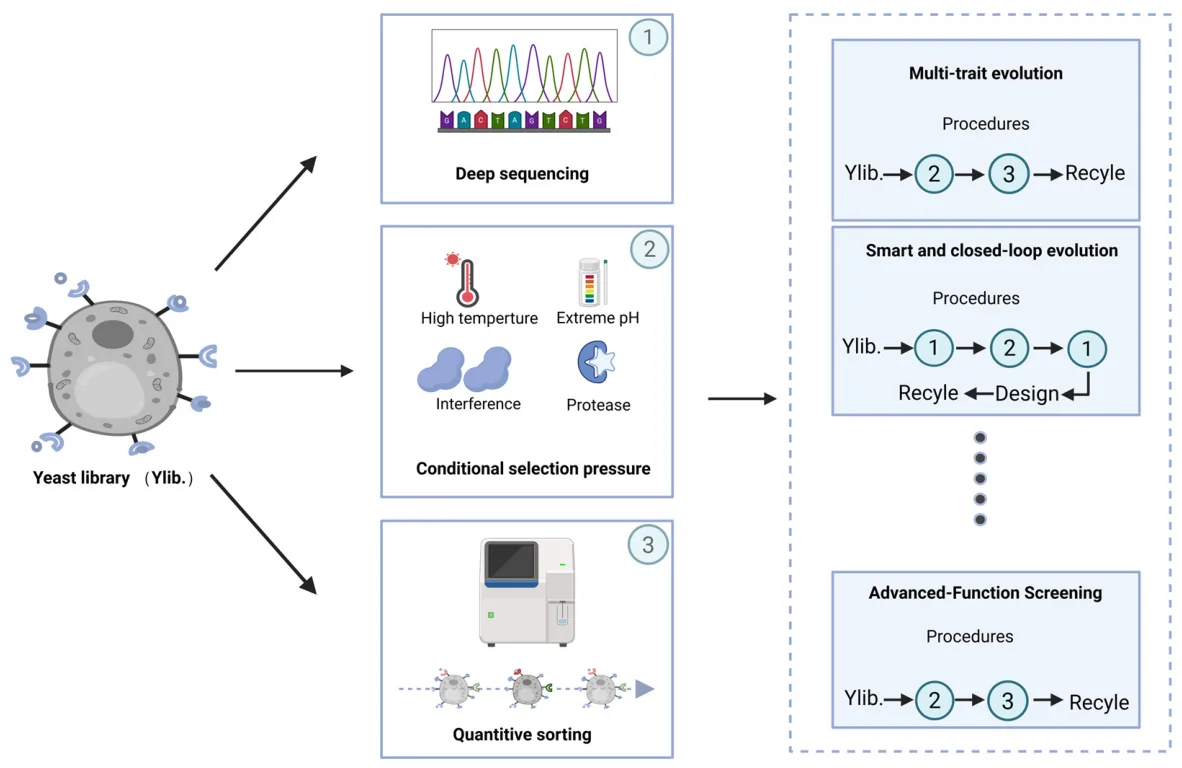
Modular integration of analytical and selection tools expands yeast surface display toward advanced evolutionary strategies. Schematic illustration of a yeast surface display platform in which the Yeast library (Ylib.) acts as a base that can be integrated with three complementary technical modules (Middle panels): (**1**) deep sequencing, (**2**) conditional selection pressure (including specific constraints such as High temperature, Extreme pH, Interference, and Protease), and (**3**) quantitative sorting (Flow cytometry). The specific coupling of these modules enables distinct evolutionary workflows (as summarized in the right-hand panel (The numbers here indicate the corresponding techniques in the middle panel.; the ellipsis “…” indicates more combination schemes). For example, combining deep sequencing (module 1 in middle panels) with conditional selection pressure (module 2 in middle panels) establishes the smart and closed-loop evolution workflow (as depicted in the second rectangle of the right panels), incorporating an additional design step into the iterative cycles.

**Figure 7:**
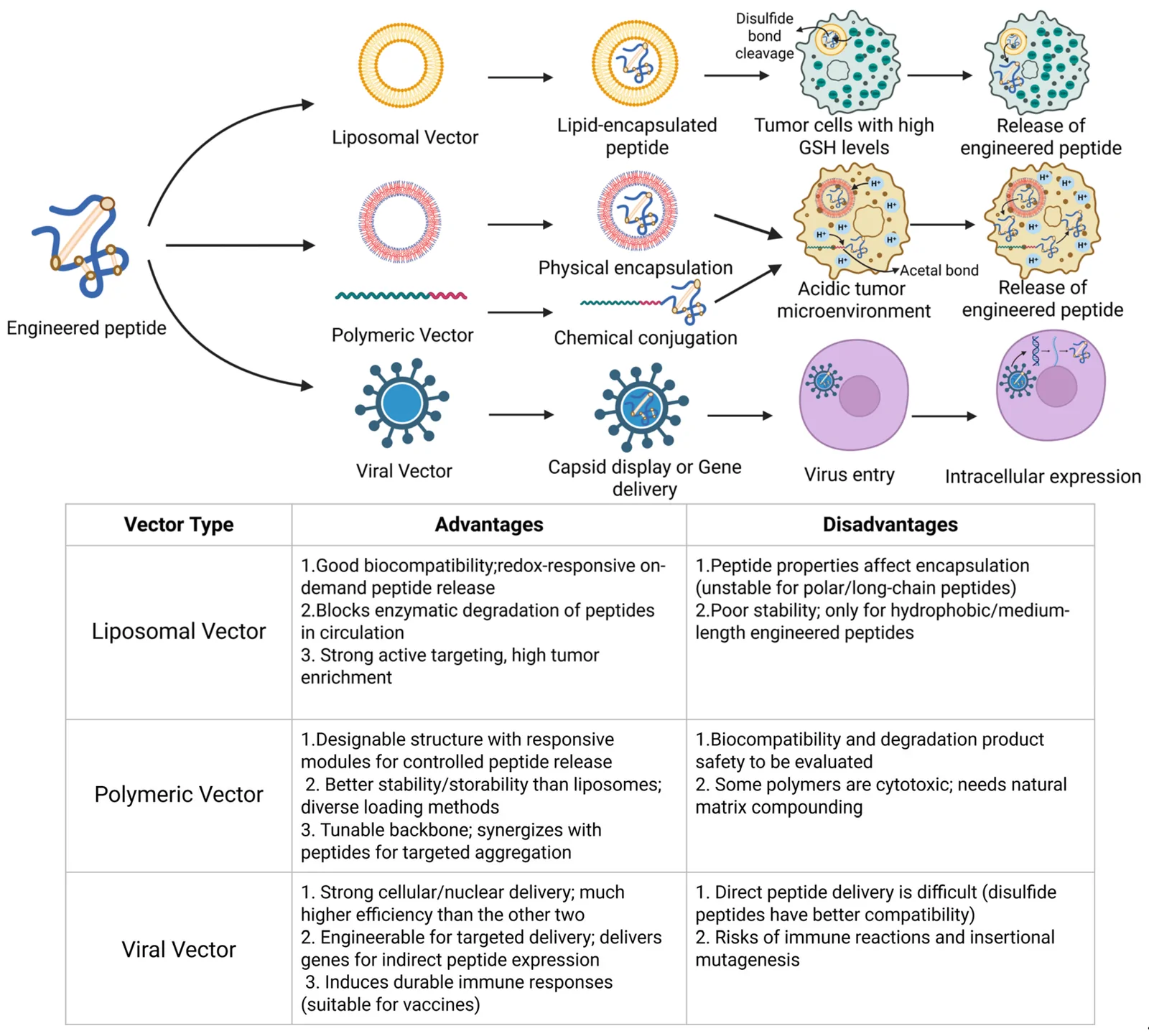
Comparative delivery and release mechanisms of engineered peptides via liposomal, polymeric, and viral vectors. Schematic illustration of three distinct vector systems for peptide delivery. The upper panel depicts the loading and release mechanisms of the engineered peptide in three different carrier platforms: the liposomal vector, the polymeric vector, and the viral vector. Liposomal vectors release the encapsulated peptide via disulfide bond cleavage in tumor cells with high GSH levels. Polymeric vectors exploit acid-labile acetal bonds for pH-triggered release in the acidic tumor microenvironment. Viral vectors mediate delivery through capsid display or gene delivery, leading to intracellular expression of the engineered peptide. The lower panel provides a comparative summary table, presenting the respective advantages and disadvantages for each vector type.

## Data Availability

All data analyzed in this review are derived from previously published literature, which can be found in the reference list of this article.
